# Supplementary material for: coiaf: Directly estimating complexity of infection with allele frequencies
Source: PLoS Comput Biol. 2023 Jun 9;19(6):e1010247. doi: 10.1371/journal.pcbi.1010247 (PMC10310041; doi:10.1371/journal.pcbi.1010247)
Supplement: S1 Appendices — Includes the complete problem formulation, including derivations of the Variant Method and the Frequency Method, additional information about our methods and software package, and all omitted figures. (PDF) [file pcbi.1010247.s001.pdf]

S1 Appendices

Contents

Appendix A Reference and minor allele frequencies **2**

Appendix B Derivation of Variant and Frequency Methods **3**
B.1 Number of strains containing the minor allele 3
B.2 Variant Method: probability a locus is heterozygous 3
B.3 Frequency Method: expected within-sample minor allele frequency 3

Appendix C Additional mathematical considerations **5**
C.1 An unbiased estimator of the PLMAF 5
C.2 Impact of correlation among loci 6

Appendix D Accounting for sequence error **7**

Appendix E Bootstrapped confidence intervals **8**

Appendix F Convergence of bootstrap replicates **9**

Appendix G Simulator details **10**
G.1 Simulator parameters 10

Appendix H Description of parameters **12**

Appendix I Benchmarks **13**

Appendix J Sampling information per region **16**

Appendix K Running *THE REAL McCOIL* on real data **17**

Appendix L Omitted figures **18**
L.1 Sensitivity analysis 18
L.1.1 Comparison to *THE REAL McCOIL* 28
L.1.2 Estimated PLMAF 31
L.2 Clustering real data samples 34
L.3 Real data 35

References **41**

Appendix A Reference and minor allele frequencies

We define the within-sample allele frequency (WSAF) as a vector, $\mathbf{w}^{\mathbf{alt}}$, of length l composed of the frequencies of the alternate allele, i.e., the non-3D7 reference, at each locus for a single individual infection. Likewise, we define the population-level allele frequency (PLAF) as a vector, $\mathbf{p}^{\mathbf{alt}}$, of length l composed of the frequencies of the alternate allele at each locus across a population. The PLAF may be represented as the mean of the WSAF across a population of size n, i.e., at locus i, $p_i^{alt} = \frac{1}{n}\sum_{j=1}^{n} w_{ij}^{alt}$.

As introduced in the main text, the population-level minor allele frequency (PLMAF) is defined as a vector, $\mathbf{p}$, of length l composed of the frequencies of the minor allele at each locus across a population. Namely $\mathbf{p} = (p_1, \ldots, p_l)$, where $p_i \in [0, 0.5]$. The within-sample minor allele frequency (WSMAF) is defined as a vector, $\mathbf{w}$, of length l composed of the frequencies of the population-level minor allele at each locus for a single individual infection. Given the WSAF and the PLAF, the WSMAF and the PLMAF at a particular locus i may be expressed as follows:

$$(w_i, p_i) = \begin{cases} (w_i^{alt}, p_i^{alt}) & p_i^{alt} \leq 0.5 \\ 1 - (w_i^{alt}, p_i^{alt}) & p_i^{alt} > 0.5 \end{cases}. \tag{1}$$

Appendix B Derivation of Variant and Frequency Methods

Let us assume there are l biallelic loci for a given individual. We define the PLMAF and WSMAF as in the main text and Appendix A.

B.1 Number of strains containing the minor allele

Let each genetically distinct haplotype in a sample be referred to as a "strain" (note that this does not necessarily imply any difference in phenotype between strains). Let k be the (unknown) number of strains in a sample, i.e., the complexity of infection (COI) of the sample. We first derive an expression for the number of strains that carry the minor allele. At locus i, let $\mathbf{C}_i = (C_{i,1}, \ldots, C_{i,k})$ be a vector of Bernoulli random variables, where $C_{i,j} = 1$ if strain j carries the minor allele, and $C_{i,j} = 0$ otherwise. Let the random variable N_i represent the number of strains that carry the minor allele, such that $N_i \in \{0, \ldots, k\}$ and $N_i = \sum_{j=1}^{k} C_{i,j}$. If we make the strong assumption that parasite strains within a mixture are unrelated, then the genetic material of each strain in a sample can be considered independently drawn from the PLMAF. Our assumption implies that

$$\mathbb{P}(C_{i,j} = 1) = p_i \,, \quad \forall j \,. \tag{2}$$

As a result, N_i will follow a Binomial distribution with a probability of success (i.e., drawing the minor allele) defined by Eq (2):

$$\mathbb{P}(N_i = n_i) = \binom{k}{n_i} p_i^{n_i} (1 - p_i)^{k - n_i} \,, \quad n_i \in \{0, \ldots, k\} \,. \tag{3}$$

We can determine the probability of strain j carrying the minor allele given that n_i strains carry the minor allele. Given n_i, we know that there must be n_i values of the vector $\mathbf{C}_i$ that equal one and $k - n_i$ that equal zero. As a result, we can express the probability that a given $C_{i,j}$ is equal to one as follows:

$$\mathbb{P}(C_{i,j} = 1 | N_i = n_i) = \frac{n_i}{k} \,. \tag{4}$$

B.2 Variant Method: probability a locus is heterozygous

In the Variant Method, we examine all loci and wish to identify the probability of locus i being heterozygous. Recall that heterozygous sites contain two distinct alleles: the major and minor alleles. We define a Bernoulli random variable, V_i, which takes the value of one if a site is heterozygous and zero otherwise. Therefore, the probability that locus i is heterozygous, written as $\mathbb{P}(V_i = 1)$, is equal to one minus the probability that a locus is homozygous, i.e., all strains contribute to the minor allele, or all strains contribute to the major allele. Using Eq (3), we write,

$$\mathbb{P}(V_i = 1) = 1 - p_i^k - (1 - p_i)^k \,. \tag{5}$$

B.3 Frequency Method: expected within-sample minor allele frequency

As before, suppose our sample contains k distinct parasite strains. For the Frequency Method, we assume that our genotyping method produces integer counts of each strain rather than simply recording the presence or absence of a strain. Let $\mathbf{s}_i = (s_{i,1}, \ldots, s_{i,k})$ represent the proportion of each strain at locus i, such that $s_{i,j} \geq 0 \,, \forall j$ and $\sum_{j=1}^{k} s_{i,j} = 1$. The within-sample minor allele frequency (WSMAF) at locus i, denoted by the random variable W_i, is defined as the sum of all strain proportions that carry the minor allele. We can, therefore, write,

$$W_i = \sum_{j=1}^{k} s_{i,j} C_{i,j} \,. \tag{6}$$

It follows from the linearity of expectation that

$$\mathbb{E}[W_i] = \sum_{j=1}^{k} s_{i,j}\mathbb{E}[C_{i,j}]\,. \tag{7}$$

By definition, as $C_{i,j}$ follows a Bernoulli distribution (Eq (2)), the expected value is equal to p_i. Moreover, as the sum over all strain proportions, $s_{i,j}$, is equal to 1,

$$\begin{aligned}\mathbb{E}[W_i] &= p_i \sum_{j=1}^{k} s_{i,j} \\ &= p_i\,.\end{aligned} \tag{8}$$

We arrive at the result that the expectation of the WSMAF is equal to the PLMAF. It follows that the mean of W_i over all individuals in a region is an unbiased estimator of p_i (subject to the caveats above). Notably, this result stands regardless of any assumptions about the distribution of strain proportions. However, this will be a biased estimator of p_i if $s_{i,j}$ are not independent of $C_{i,j}$. For instance, if $\mathbf{C}_i$ is associated with drug resistance and sample collection was biased to reveal this non-independence, i.e., samples were collected from individuals after drug treatment, increasing resistant parasite strains, our estimator would differ between the treated and untreated malaria populations. This finding is further explored in Appendix C.

We may also derive the expectation of W_i given that locus i is heterozygous (i.e., $V_i = 1$). We first find the expectation of W_i given that n_i strains carry the minor allele using the linearity of conditional expectation. Applying the same logic as when deriving Eq (8), we know that the expected value of Eq (4) is equal to $\frac{n_i}{k}$, by definition, and that the sum of all strain proportions is equal to 1. Therefore,

$$\begin{aligned}\mathbb{E}[W_i|N_i = n_i] &= \sum_{j=1}^{k} s_{i_j}\mathbb{E}[C_{i,j}|N_i = n_i] \\ &= \frac{n_i}{k}\,.\end{aligned} \tag{9}$$

We then leverage the law of total expectations:

$$\mathbb{E}[W_i|V_i = 1] = \sum_{n_i=0}^{k} \mathbb{E}[W_i|V_i = 1, N_i = n_i]\mathbb{P}(N_i = n_i|V_i = 1)\,. \tag{10}$$

We note that if $V_i = 1$, this implies that $0 < N_i < k$. As a result,

$$\mathbb{E}[W_i|V_i = 1] = \sum_{n_i=1}^{k-1} \mathbb{E}[W_i|N_i = n_i]\frac{\mathbb{P}(N_i = n_i)}{\mathbb{P}(V_i = 1)}\,. \tag{11}$$

Substituting in Eqs (3), (5), and (9), we find that

$$\begin{aligned}\mathbb{E}[W_i|V_i = 1] &= \sum_{n_i=1}^{k-1} \frac{n_i}{k}\frac{\binom{k}{n_i}p_i^{n_i}(1-p_i)^{k-n_i}}{1-p_i^k-(1-p_i)^k} \\ &= \frac{k^{-1}}{1-p_i^k-(1-p_i)^k}\sum_{n_i=1}^{k-1} n_i\binom{k}{n_i}p_i^{n_i}(1-p_i)^{k-n_i}\,.\end{aligned} \tag{12}$$

We note that the sum in Eq (12) represents the expected value of the binomial distribution (equal to kp_i) less the case where $n_i = 0$ (equal to 0) or $n_i = k$ (equal to kp_i^k). Therefore, after some algebra, we obtain

$$\mathbb{E}[W_i|V_i = 1] = \frac{p_i - p_i^k}{1-p_i^k-(1-p_i)^k}\,. \tag{13}$$

Note that the Variant and Frequency Methods depend on the COI and the PLMAF. However, the Variant Method identifies the probability of a locus being heterozygous, denoted as $\mathbb{P}(V_i = 1)$. In contrast, the Frequency Method identifies the expected value of the WSMAF given a site is heterozygous, denoted as $\mathbb{E}[W_i|V_i = 1]$.

Appendix C Additional mathematical considerations

C.1 An unbiased estimator of the PLMAF

In Appendix B.3, we derived the expression

$$\mathbb{E}[W_i] = p_i \,, \tag{8}$$

and commented that the mean of W_i over all individuals in a region serves as an unbiased estimator of p_i. In particular, regardless of any assumptions about the distribution of strain proportions and COIs for a set of samples, the mean WSMAF will equal the PLMAF on average. We note that this assumes no sequencing error and that sample collection is independent of strain proportions. To further illustrate that this result stands regardless of the distribution of COIs, we simulated the WSMAF for several samples with different COIs and compared the mean WSMAF over all individuals to the true PLMAF. We first generated a random set of samples with different COIs. For each sample, we found $\mathbf{C}_i$ by sampling from a Bernoulli distribution and $\mathbf{s}$ by assuming that strain proportions were sampled from a symmetric Dirichlet distribution with shape parameter α, without loss of generality. Lastly, we determined the WSMAF for each sample by using Eq (6) and found the mean WSMAF. Simulations were run with different numbers of samples, different COI values, and different values of the shape parameter. The results are illustrated in Fig A, confirming that the true PLMAF is recovered by averaging the population observed WSMAF.

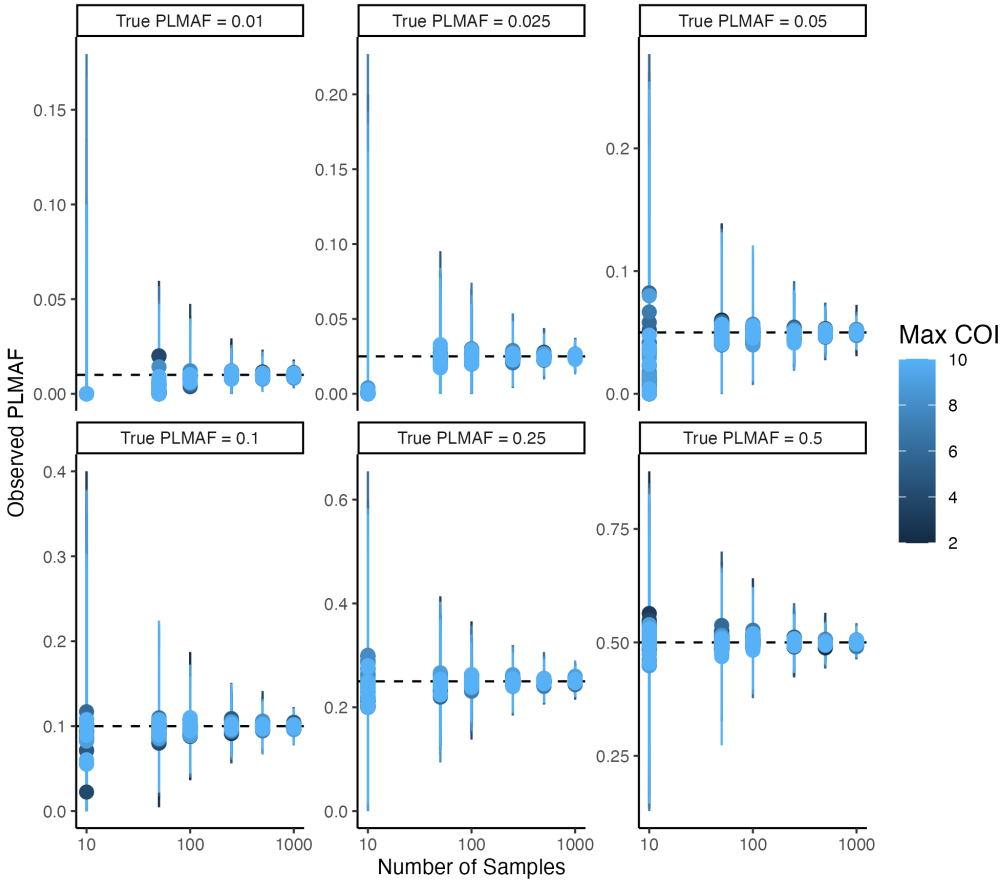

Fig A. Observed PLMAF based on WSMAF. The observed PLMAF was simulated using different parameters. For each set of parameters, data was simulated 50 times. Each point represents the mean observed PLMAF, and the bars represent the 95% quantile range. The true PLMAF is indicated by a dashed horizontal black line.

C.2 Impact of correlation among loci

In Appendix B, Eq (5), we derived the probability that a locus is heterozygous, which relied on the assumption that the genetic material contributing to the locus is independently drawn from the PLMAF. This is the same assumption that the *THE REAL McCOIL* assumes, as does the earlier model, *COIL*, on which it is based. This is because dependence between loci, i.e., linkage disequilibrium (LD), violates the assumption that each locus solely depends on allele frequencies and can thus be described with a Bernoulli process.

If loci are not independent, the probability distribution describing the number of strains containing the minor allele at a locus can no longer be described using a Binomial distribution as in Appendix B, Eq (3). If any locus is in LD, the number of strains that carry the minor allele will also depend on the degree of LD and the genotype of each strain at neighboring sites in LD. For example, if two loci are correlated due to LD, and if one locus returns a polymorphic call, the other locus is more likely to be polymorphic. However, the opposite is true: if one locus is homozygous, the probability that the other locus will also be homozygous increases. Consequently, with very large numbers of loci, these antagonistic effects will cancel each other out, and our estimator will remain unbiased.

The variance in our estimator, on the other hand, will increase as we do not account for the fact that non-independent loci are less informative than independent loci. In Fig B, we compare the distribution of point estimates for two data sets across several COIs. Data were sampled from 1,000 independent loci to generate the first data set. To generate the second data set, we first sampled from 500 independent loci and subsequently duplicated these data points—yielding a set of 1,000 dependent loci. We observe that the variance in estimated COI is smaller when sampling from the set of independent loci.

This same finding also extends to the Frequency Method as follows. The expectation of the Correlated Binomial Distribution is also equal to np. Consequently, we can make the same substitution in creating Eq (13), replacing the sum in Eq (13) with np. The denominator in Eq (13) is given by Eq (5), which we have previously detailed will not impact the bias of our method but will impact the variance of our estimator.

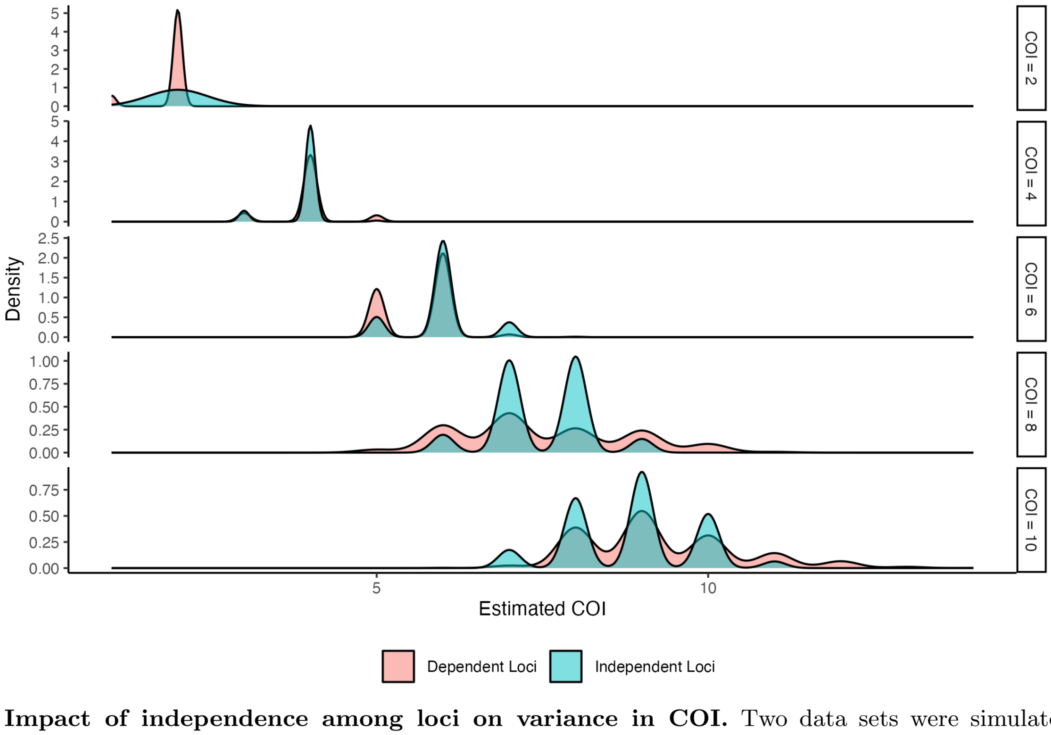

Fig B. Impact of independence among loci on variance in COI. Two data sets were simulated across varying COIs from 2 to 10. Data was simulated 10 times for each COI, and 1,000 bootstrap replicates were run to generate a distribution of point estimates. The first data set, "Independent Loci" (shaded blue), was sampled from 1,000 independent loci. To generate the second data set, "Dependent Loci" (shaded red), we first sampled from 500 independent loci. These 500 loci were then duplicated to yield 1,000 dependent loci. The title of each subplot, denoted on the rightmost side of the figure, indicates the COI that data were sampled with.

Appendix D Accounting for sequence error

During sequencing, errors may arise for several reasons. The major next-generation sequencing technologies use a sequencing-by-synthesis method where each DNA molecule in a sample is amplified individually (e.g., spots on a chip) prior to sequencing. Early errors in this amplification process can lead to called errors in the sequence. Moreover, errors may occur during the actual sequencing. To provide a template for the synthesis of the complementary strand, many copies of the same DNA molecule are on each spot. On each synthesis cycle, a new nucleotide is added to all of the growing DNA strands. However, there is a small chance that a nucleotide will not be added or more than one nucleotide will be added to some of the strands within a spot. These strands are considered "out-of-phase." As the number of cycles increases, the proportion of these "out-of-phase" strands increases, which leads to mistakes in the final read-out.

To address these errors, our software accounts for sequencing errors. The user has two options to do so. The first option allows the user to specify the amount of error believed to be present in their samples, giving complete control over the level of error assumed to the user. For instance, the user may specify a 5% sequencing error. Note that this is the default approach taken in our software, and our package, by default, assumes a 1% sequencing error.

The second option uses an algorithm to infer the sequencing error from the distribution of the input data. In particular, we focus on data with a low PLMAF and compare the true number of data points with a WSMAF greater than zero to the expected number of points with a WSMAF greater than zero. Consider an arbitrary data point (p_i, w_i). We define a partition over the range of p_i constituent of sets $\mathcal{P}_1, \ldots, \mathcal{P}_N$ such that $p_i \in \mathcal{P}_m$ if $p_i \in [a_m, b_m), \quad a_m \leq b_m,\ i = 1, \ldots, l$. In doing so, we group our data into N bins. We first count the number of loci in the first bin. We define the PLMAF for this partition as follows:

$$\hat{p}_m = \frac{a_m + b_m}{2}. \tag{14}$$

Given the number of loci in the first partition and the PLMAF, we can compute the expected number of loci containing the minor allele. Recall that sampling the minor allele at a locus can be expressed using a Binomial distribution with one trial and a probability of success (drawing the minor allele) equal to the PLMAF at that locus. Therefore, to find the expected number of loci with the minor allele, we can multiply the number of loci with the probability of a locus being the variant allele. As the PLMAF tends towards zero, we expect very few sites to have the minor allele. Therefore, if we remove the expected number of loci from the true number of loci with a minor allele, all the remaining loci will likely result from sequencing error. By examining the distribution of the WSMAF of these points, we can estimate the amount of sequence error present in our sample. If this sequence error is small, we enforce a minimum sequencing error of 1%.

Following the determination of the level of sequencing error, ϵ, thought to be present in the data, our algorithms account for the error as follows. Note that we assume that sequencing error affects the probability of correctly sampling both the minor and the major alleles. Therefore, loci at which $0 < \text{WSMAF} \leq \epsilon$ and loci at which $1 > 1 - \text{WSMAF} \geq 1 - \epsilon$ are assumed to have been incorrectly sampled and are denoted as homozygous loci instead of heterozygous loci.

Appendix E Bootstrapped confidence intervals

To provide a confidence interval for our COI estimates, we use the boot package in R [2]. To do so, we randomly sample with replacement from our set of data points ($D : \{(p_i, w_i, d_i), \quad i = 1, \ldots, l\}$) multiple times and determine the estimated COI for each replicate. From this distribution, we then determine the 95% confidence interval. The default settings for this process sample with replacement 100 times (see Appendix F for additional information on this default value). Furthermore, as bootstrapping may be a computationally expensive technique, we have provided options for users to parallelize the process using multiple CPUs. An illustration of the distribution resulting from bootstrapping on a set of simulated data is shown in Fig C.

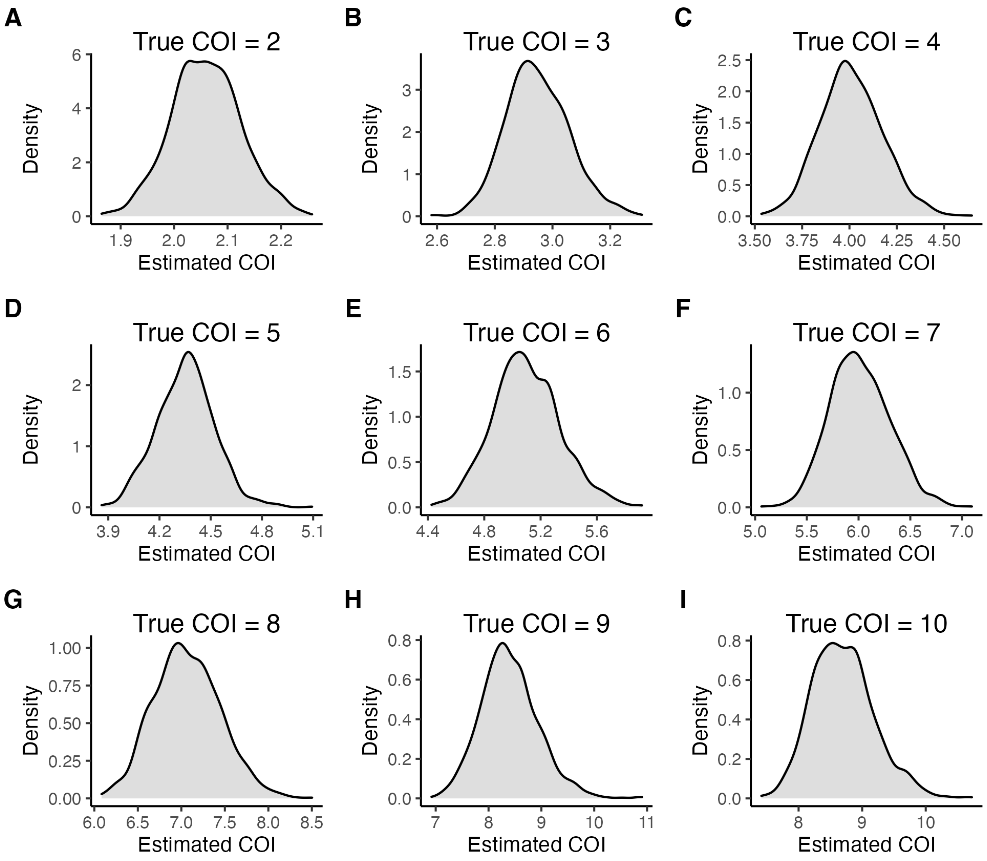

Fig C. Bootstrapped confidence intervals across a range of COIs. The kernel density estimate of the distribution is plotted for simulated data. Data were sampled with 1,000 loci from a range of COIs from two to ten.

Appendix F Convergence of bootstrap replicates

As discussed in Appendix E, we leverage bootstrapping to provide users with a confidence interval for COI estimates. To do this, we sample with replacement multiple times and determine the estimated COI for each replicate. When estimating the confidence interval using this strategy, it is important to have a sufficiently large number of replicates such that increasing the number will not meaningfully alter the variance in COI estimates. In Fig D, we explore how changing the number of replicates alters the standard error when estimating the COI on simulated data.

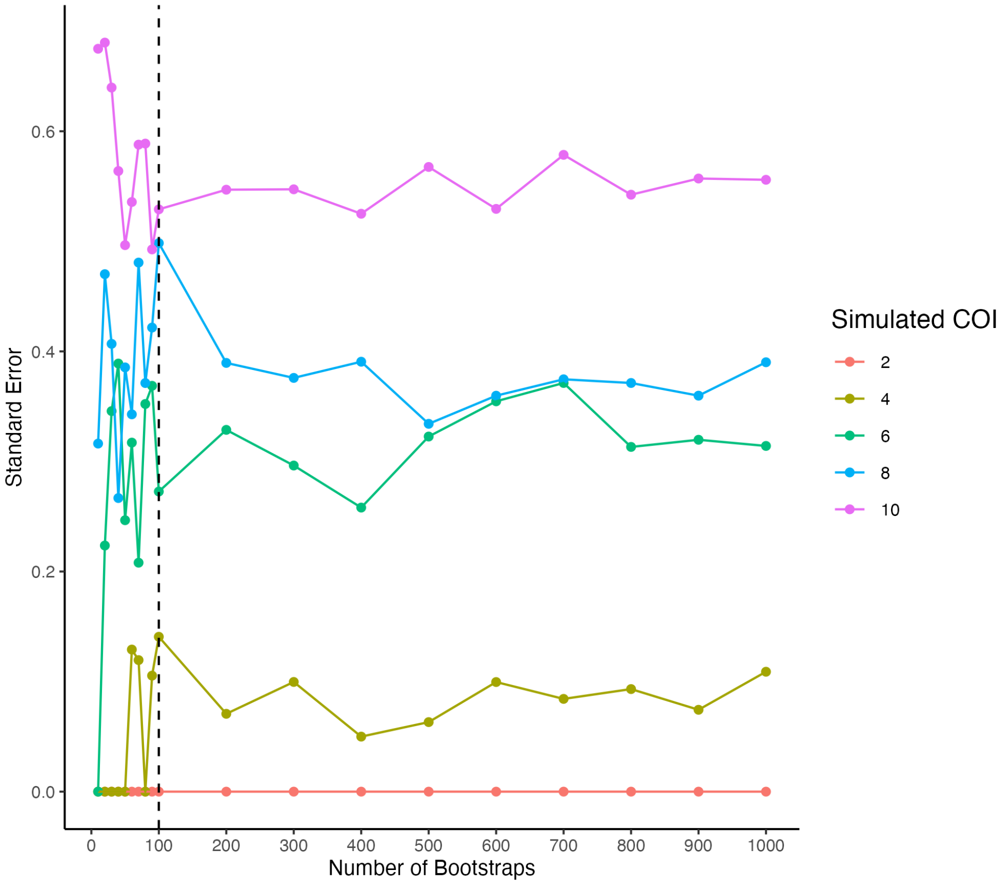

Fig D. Standard error in estimates across replicates. Data were simulated from a simple simulation with 1,000 loci and a COI ranging from 2 to 10. For each simulation, the COI and confidence interval was estimated, and the standard error was plotted along the y-axis for an increasing number of replicates (x-axis).

Fig D illustrates that the variance decreases as the number of replicates increases. We note that the variance with 100 replicates is largely comparable to the variance with 1,000 replicates. Given this, we set the default number of replicates to 100 in our software package.

Appendix G Simulator details

To test our mathematical methods, we created a simulator that generates synthetic sequencing data for an individual in a given population. The individual is assigned a COI value, which is used to simulate the number of sequence reads mapped to the reference and alternative alleles for the biallelic SNPs considered. These are then used to derive the within-sample frequency of the population-level minor allele, w_i, at each locus.

We first define the number of loci simulated, l. We assume that the distribution of reference allele frequencies for locus i, R_i, is described by a Beta distribution with shape parameters α and β:

$$\mathbb{P}(R_i = r_i) = \frac{\Gamma(\alpha+\beta)}{\Gamma(\alpha)\Gamma(\beta)} r_i^{(\alpha-1)}(1-r_i)^{(\beta-1)} \,,$$

where Γ is the Gamma function. In our simulations, we assume $\alpha = 1$ and $\beta = 5$ and draw l values of R_i. In Eq (5) and (13), we have defined relationships with respect to the frequency of the minor allele, p_i. Recall that $p_i \in [0, 0.5]$. Thus, we define

$$p_i = \begin{cases} r_i & r_i \leq 0.5 \\ 1 - r_i & r_i > 0.5 \end{cases} .$$

To simulate w_i for each individual, we first assign the COI, k. Next, we simulate the genotype at locus i, drawing k values from a Binomial distribution with probability p_i, i.e., the probability of having the minor allele at locus i is equal to the population-level frequency of the minor allele. This yields the matrix, $\mathbb{G} \in \mathbb{R}^{k \times l}$, with k rows and l columns defining the phased haplotype of each strain, where $\mathbb{G}_{i,j} = 1$ indicates that strain j at locus i has the minor allele.

To determine the number of sequence reads related to each strain, we first draw the proportion of each strain, $\mathbf{S} = (S_1, \ldots, S_k)$, in an individual with k strains, which we assume is described by a Dirichlet distribution with concentration parameters $\alpha_1, \ldots, \alpha_k$. The "true" WSMAF of the minor allele at locus i, denoted τ_i, is thus given by the sum of the strain proportions for strains with the minor allele. We can express this using matrix multiplication:

$$\boldsymbol{\tau} = \mathbf{S} \times \mathbb{G} \,,$$

where $\boldsymbol{\tau} \in \mathbb{R}^{1 \times l}$, $\mathbf{S} \in \mathbb{R}^{1 \times k}$, and $\mathbb{G} \in \mathbb{R}^{k \times l}$.

In simulations with no assumed sequence error, the number of sequence reads with the minor allele at each locus is given by sampling from a Binomial distribution c times with probability $\boldsymbol{\tau}$, where c is the assumed number of sequence reads, i.e., the read depth or coverage. However, in simulations with sequence error, we perturb the "true" WSMAF by assuming that a number of sequence reads are incorrectly sequenced. An incorrectly sequenced locus with the major allele will yield the minor allele and vice versa. In our simulations, we assume a fixed sequence error, ϵ, such that the probability of correctly sequencing the minor allele is $1 - \epsilon$, and the probability of incorrectly sequencing the minor allele is ϵ. Therefore, we can represent the probability of sequencing the minor allele, now denoted as $\boldsymbol{\tau}_\epsilon$ to indicate the added error, as

$$\boldsymbol{\tau}_\epsilon = \boldsymbol{\tau}(1-\epsilon) + (1-\boldsymbol{\tau})\epsilon \,.$$

In simulations with relatedness assumed, we sample loci from other strains in the mixed infection with a probability equal to the relatedness simulated. Lastly, we may account for overdispersion—additional unexpected variability in our data—and sample the number of sequence reads with the minor allele at each locus from a Beta-binomial distribution rather than a Binomial distribution. Dividing the number of instances of the minor allele by the coverage at each locus results in the simulated WSMAF.

G.1 Simulator parameters

As previously mentioned, in our simulator, we assume that a Beta distribution describes the distribution of the reference allele frequencies for a given locus. In addition, we account for overdispersion in parasite densities. In Fig E, we illustrate how these two parameters impact the probability that strains in mixed infection will contribute to the observed sequence reads.

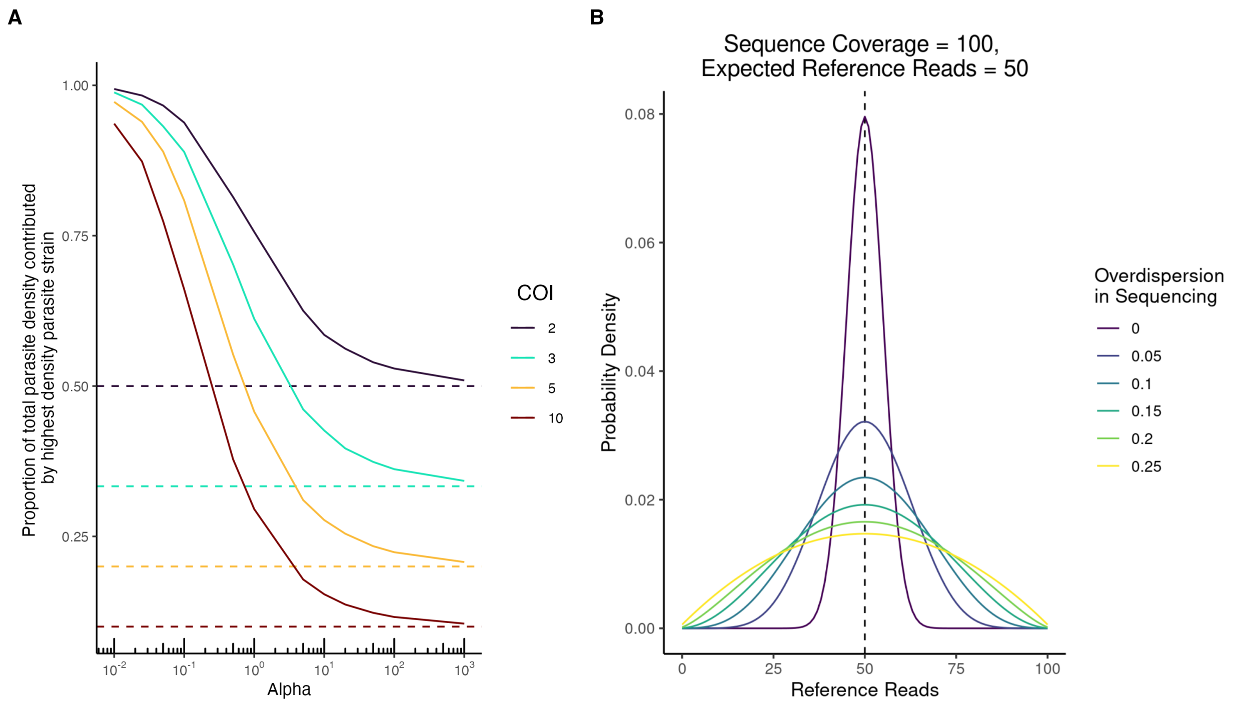

Fig E. The effect of alpha and overdispersion. (**A**) The proportion of total parasite density contributed by the highest-density parasite strain is plotted against a changing alpha parameter. The alpha parameter controls for the overdispersion in parasite densities. The dashed lines indicate the maximum parasite density if all strains had the same parasite density, i.e., 1/COI. (**B**) The number of reference reads is plotted with varying levels of overdispersion in sequencing error. Data were simulated with a sequence coverage of 100.

Appendix H Description of parameters

Parameter	Description	Default Value
Coverage	The coverage at each locus.	200
Loci	The number of loci examined across the genome.	1,000
Alpha	The alpha parameter used to generate strain proportions from a Dirichlet distribution.	1
Overdispersion	The extent to which counts are overdispersed relative to the Binomial distribution.	0
Epsilon	The probability of a single read being miscalled as the other allele. This parameter defines the level of error used when simulating data.	0
COI	The complexity of infection.	
Sequence Error	The level of sequencing error that is assumed in our estimations. For instance, if it is believed that in the population there is a 1% of sequencing error, then this parameter would be set to 0.01.	1%

Table A. Description of select parameters. The description and the default value for several important parameters are described.

Appendix I Benchmarks

To benchmark our software, we used the bench package, available in R, which tracks execution time, memory allocation, and garbage collection [3]. We conducted internal comparisons, evaluating the speed of each of our estimation and solution methods. For all internal comparisons, we simulated data with a variable number of loci: 100, 1,000, 5,000, 10,000, and a variable complexity of infection: 1, 5, 10, 15, 20, 25. We then compared the speed of our methods by running each method 100 times and plotting the time for each iteration to run. Our results are shown in Fig F and G.

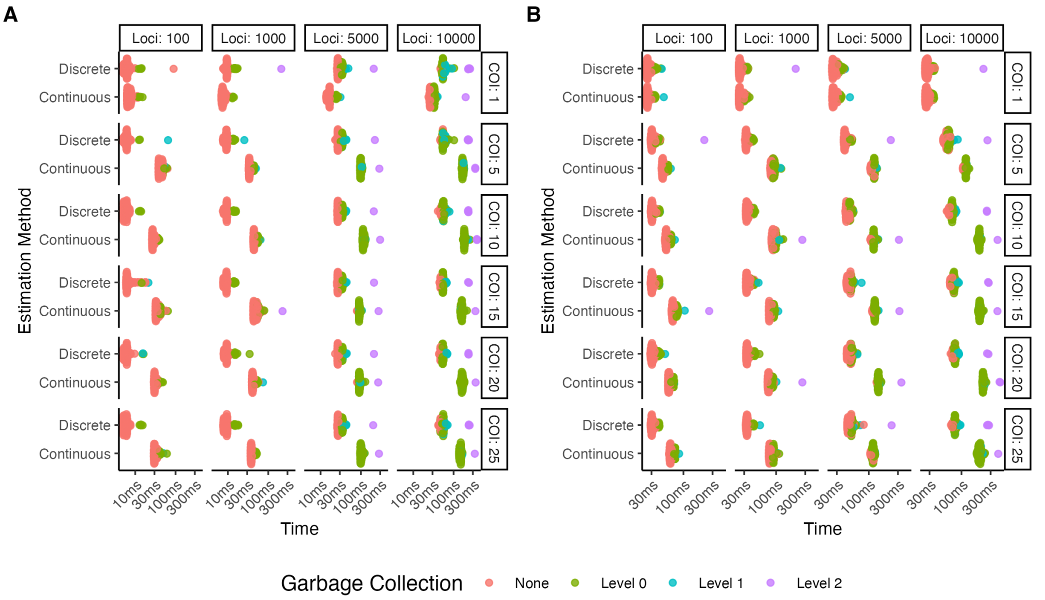

Fig F. Variant Estimation Method vs. Frequency Estimation Method. A beeswarm plot [4] was generated for the Variant Method (**A**) and the Frequency Method (**B**). For each method, we measured the speed of our algorithms with both the discrete and continuous estimation methods for a varying number of loci and COI. The color of each point represents the number of garbage collections conducted for the specific iteration.

There was little difference between our two estimation methods and two solutions methods. To evaluate whether differences between methods were statistically significant, we utilized the Wilcoxon signed-rank test, a nonparametric hypothesis test [5]. In both the Variant Method and the Frequency Method, the discrete solution method was significantly faster than the continuous solution method (p-value: < 0.001 in both cases). Of the Variant and the Frequency Methods, the Variant Method was significantly faster than the Frequency Method (p-value: < 0.001).

We additionally ran benchmarks to test our software package against the current "state-of-the-art" method, *THE REAL McCOIL*. All benchmarks were run ten times to reduce variance between runs. We simulated data with a COI from 1 to 10 and compared the speed of our different estimation methods to the speed of *THE REAL McCOIL*. The *THE REAL McCOIL* was run with a burn-in period of $2,000$ followed by $5,000$ sampling iterations. These numbers were chosen by simulating a complex data set, running the *THE REAL McCOIL* on it with different parameter values, and examining the convergence between Monte Carlo Markov chains [6]. We found that even with a complex data set, convergence was met with a burn-in period of $2,000$ followed by $5,000$ sampling iterations. Fig H shows the median run time across the ten runs across a varying number of loci and samples.

Comparing our various estimation methods to *THE REAL McCOIL*, we observe that the speed of the point estimates generated by the discrete and continuous Variant Methods of *coiaf* remains constant even as the number of loci increases. All other evaluated estimation methods increase in a seemingly linear fashion. Importantly, the rate at which the run time of our methods increases is substantially lower than the rate at which the run time of *THE REAL McCOIL* increases. Depending on which estimation method the user chooses, our methods will be faster than *THE REAL McCOIL* for analyses evaluating greater than 1,000 loci.

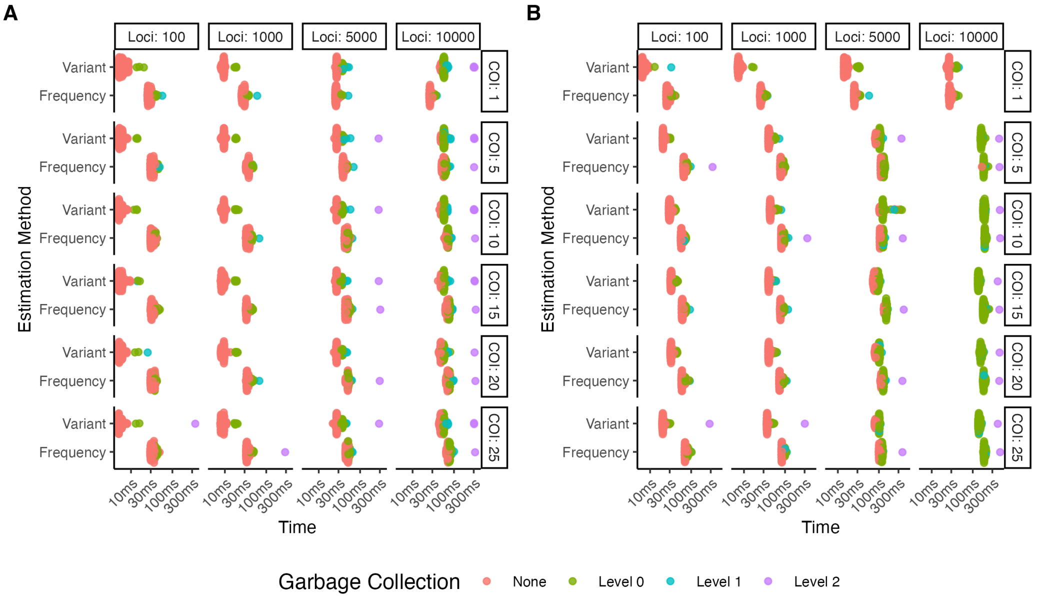

Fig G. Variant Estimation Method vs. Frequency Estimation Method. A beeswarm plot [4] was generated for the discrete solution method (**A**) and the continuous solution method (**B**). For each solution method, we measured the speed of our algorithms with the Variant Method and the Frequency Method for a varying number of loci and COI. The color of each point represents the number of garbage collections conducted for the specific iteration.

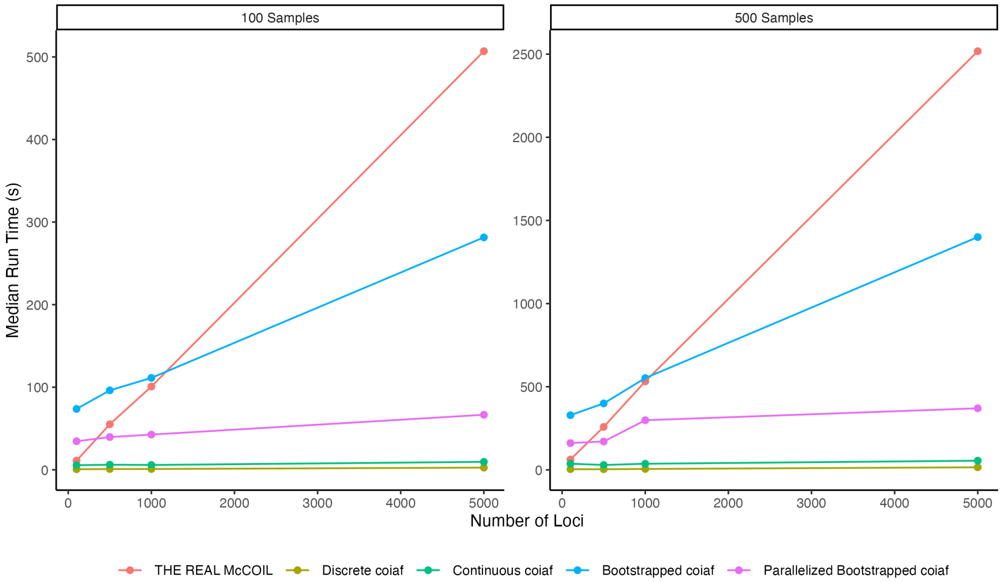

Fig H. Speed of different methods. Data were simulated with a varying number of loci and samples. For each iteration, the median run time in seconds of several estimation methods was compared. These included: *THE REAL McCOIL*, the discrete and continuous Variant Methods of *coiaf*, the bootstrapped discrete Variant Method of *coiaf*, and the parallelized bootstrapped discrete Variant Method of *coiaf*. For the bootstrapped methods, 100 replicates were used, and, in the case of the parallelized estimation, 8 CPUs were utilized.

We note that for a fair comparison of our bootstrapped methods and *THE REAL McCOIL*, it is important to illustrate that the confidence intervals between the two software packages are comparable. Fig I shows the distribution of point estimates for the two packages across COI values ranging from 2 to 10. We observe that there is little difference between the two distributions.

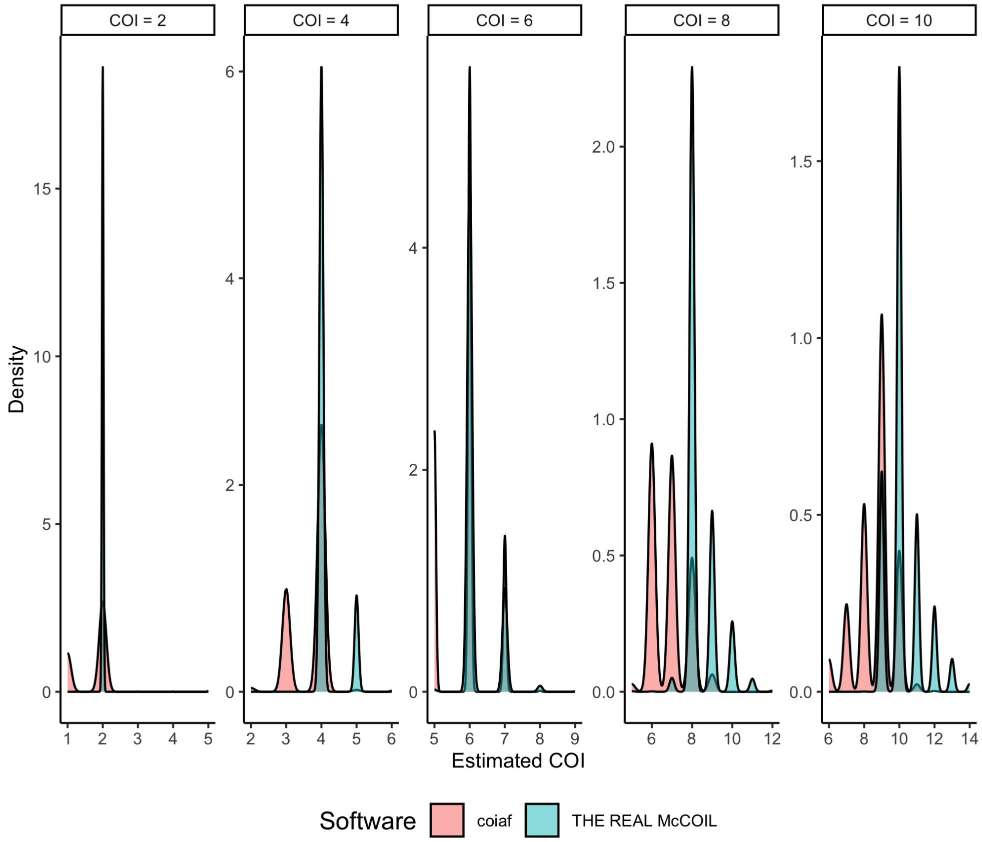

Fig I. Distribution of estimated COI. Data were simulated from 1,000 loci 10 times with a COI of 2 to 10 and a coverage of 200. The bootstrapped discrete Variant Method was used to estimate the COI for *coiaf*. The distribution of point estimates for *THE REAL McCOIL* and *coiaf* is compared. The title of each subplot indicates the COI that data were simulated with. The x-axis indicates the estimated COI, and the y-axis indicates the density.

All benchmarks were run on a standard 2019 MacBook Pro with a 2.3 GHz 8-Core Intel Core i9 processor and a 16 GB 2667 MHz DDR4 memory module.

Appendix J Sampling information per region

Region	Number of Samples	Number of Loci	Average Coverage Per Sample
1	475	40,220	89.2
2	122	35,486	92.9
3	200	38,186	80.0
4	149	38,491	88.7
5	248	37,252	53.7
6	147	37,700	46.5
7	909	31,324	80.3
8	164	28,114	73.3
9	70	26,027	62.2
10	88	37,270	79.1
11	813	40,272	71.4
12	237	38,882	85.8
13	558	27,015	40.9
14	314	31,968	54.6
15	80	18,649	70.6
16	256	38,593	48.5
17	92	24,438	91.8
18	29	19,315	90.1
19	37	15,276	56.0
20	77	34,467	70.4
21	226	30,403	102.9
22	100	30,685	51.8
23	344	38,842	43.3
24	235	37,815	62.3

Table B. Sampling information for each region. The information for each region includes the number of samples studied and the number of loci examined. Moreover, the average coverage per sample is provided.

Appendix K Running ***THE REAL McCOIL*** on real data

To compare our method against the "gold standard" for estimating the COI, *THE REAL McCOIL*, we first had to estimate the COI using *THE REAL McCOIL* [7]. The total number of variants across the genome is computationally untractable for *THE REAL McCOIL* and is unsuitable for the algorithm given that many SNPs will be in linkage disequilibrium (LD), the non-random association of alleles with one another at different loci in a given population. In response, we first identified a common set of SNPs such that the minor allele frequency of variants was greater than 0.005 globally and within each of the 24 identified regions. From this set of SNPs, we selected subsets of SNPs that were not in LD by first calculating the genetic autocorrelation [8] among SNPs by genetic distance and filtering to sets of SNPs with an autocorrelation less than 0.8. For each of the 24 regions, five unique sets of LD-filtered variants were created. The COI was estimated for each set using *THE REAL McCOIL* (v2) categorical method. Heterozygous loci were determined using the genotype called during variant calling. Default priors were assigned for each parameter used by *THE REAL McCOIL*, with a maximum observable COI equal to 25 and sequencing measurement error estimated along with COI and allele frequencies. We performed 5 repetitions for each sample, with a burn-in period of $1,000$ followed by $5,000$ sampling iterations and using standard methodology to confirm convergence between Monte Carlo Markov chains [6].

Appendix L Omitted figures

L.1 Sensitivity analysis

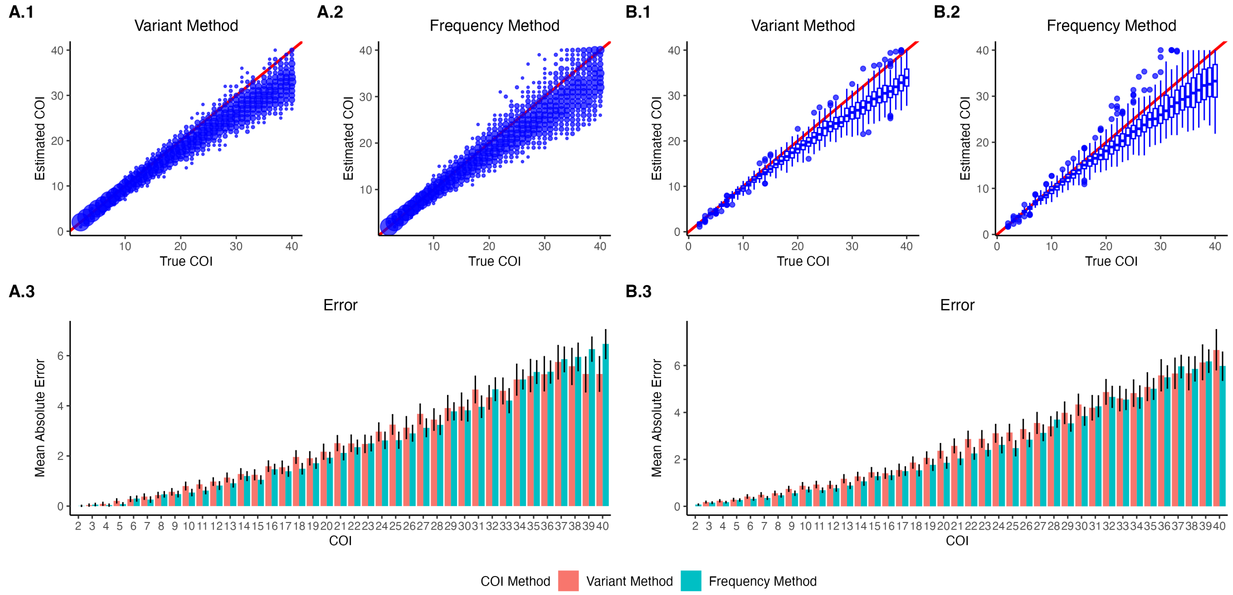

Fig J. Performance with changing COI. The performance of the Variant Method and the Frequency Method is compared across a COI of 2-40. Data was simulated 100 times from 1,000 loci with a coverage of 200. No error was introduced into our simulations, and no sequencing error was assumed. In all **A** subplots, the discrete estimation is shown, and in all **B** subplots, the continuous estimation is shown. The distribution of estimated COI for the Variant Method (**A.1**, **B.1**) and the Frequency Method (**A.2**, **B.2**) is plotted. In **A.1** and **A.2**, dot plots are used where point size indicates density. In **B.1** and **B.2**, a boxplot shows the interquartile range, with whiskers indicating the range and outliers indicated by dots. The mean absolute error is shown in **A.3** and **B.3**, with the 95% confidence interval computed from 1,000 bootstrap repetitions at each COI indicated with error bars.

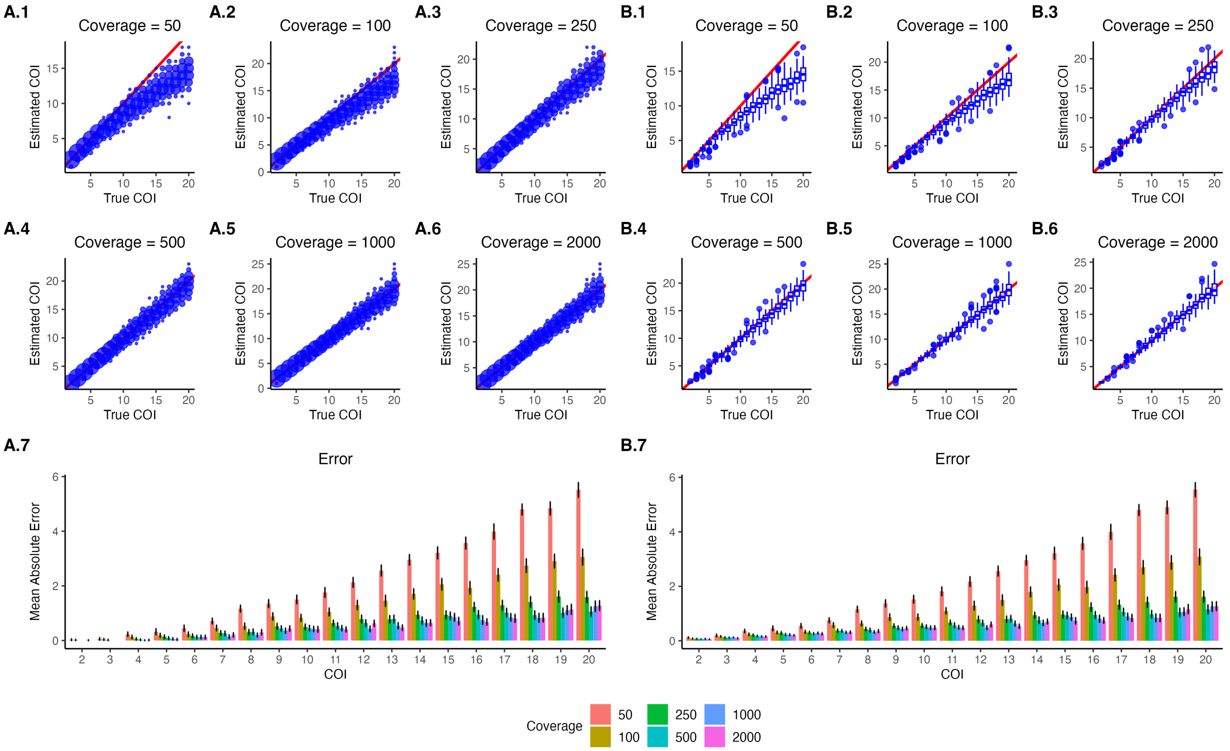

Fig K. Performance of the Variant Method with changing coverage. The performance of the Variant Method is compared across a COI of 2-20. Data was simulated 100 times with 1,000 loci. No error was introduced into our simulations, and no sequencing error was assumed. In all **A** subplots, the discrete estimation is shown, and in all **B** subplots, the continuous estimation is shown. In both cases, the distribution of the estimated COI as the coverage changes from 50 to 1,000 is shown. In **A.1**-**A.6**, dot plots are used where point size indicates density. In **B.1**-**B.6**, a boxplot shows the interquartile range, with whiskers indicating the range and outliers indicated by dots. The mean absolute error is shown in **A.7** and **B.7**, with the 95% confidence interval computed from 1,000 bootstrap repetitions at each COI indicated with error bars.

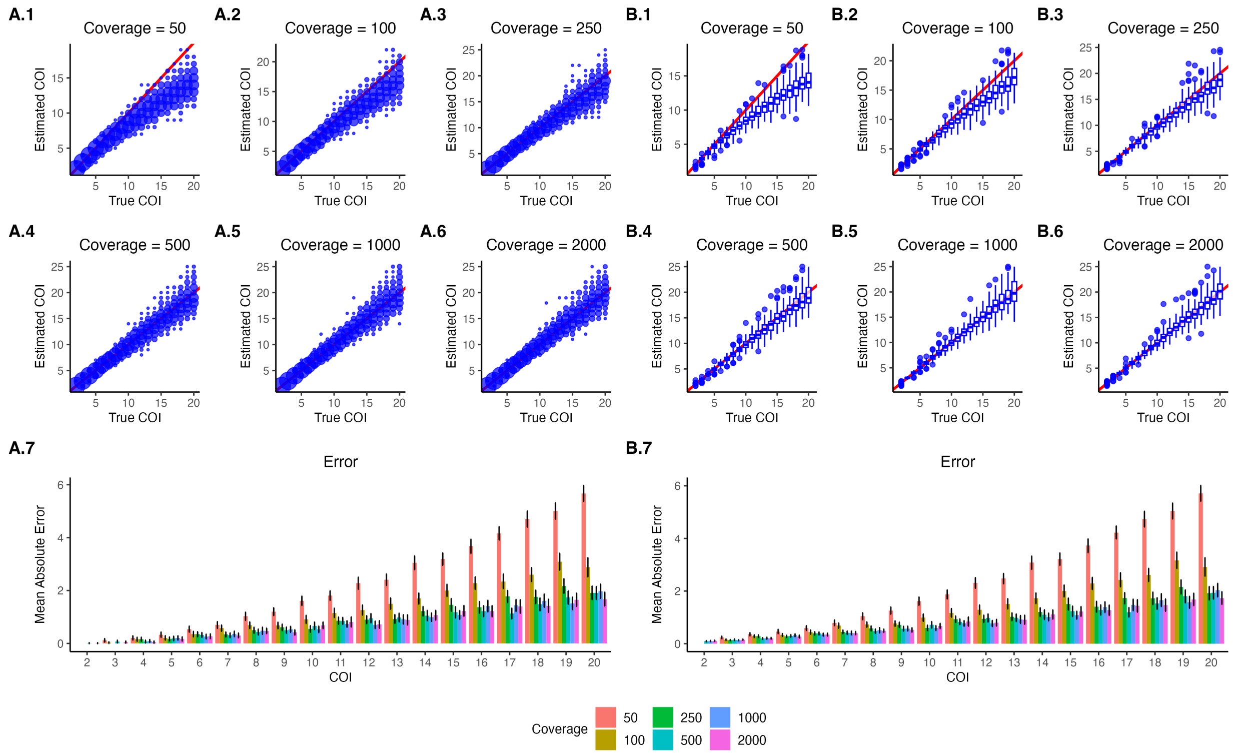

Fig L. Performance of the Frequency Method with changing coverage. The performance of the Frequency Method is compared across a COI of 2-20. Data was simulated 100 times with 1,000 loci. No error was introduced into our simulations, and no sequencing error was assumed. In all **A** subplots, the discrete estimation is shown, and in all **B** subplots, the continuous estimation is shown. In both cases, the distribution of the estimated COI as the coverage changes from 50 to 1,000 is shown. In **A.1-A.6**, dot plots are used where point size indicates density. In **B.1-B.6**, a boxplot shows the interquartile range, with whiskers indicating the range and outliers indicated by dots. The mean absolute error is shown in **A.7** and **B.7**, with the 95% confidence interval computed from 1,000 bootstrap repetitions at each COI indicated with error bars.

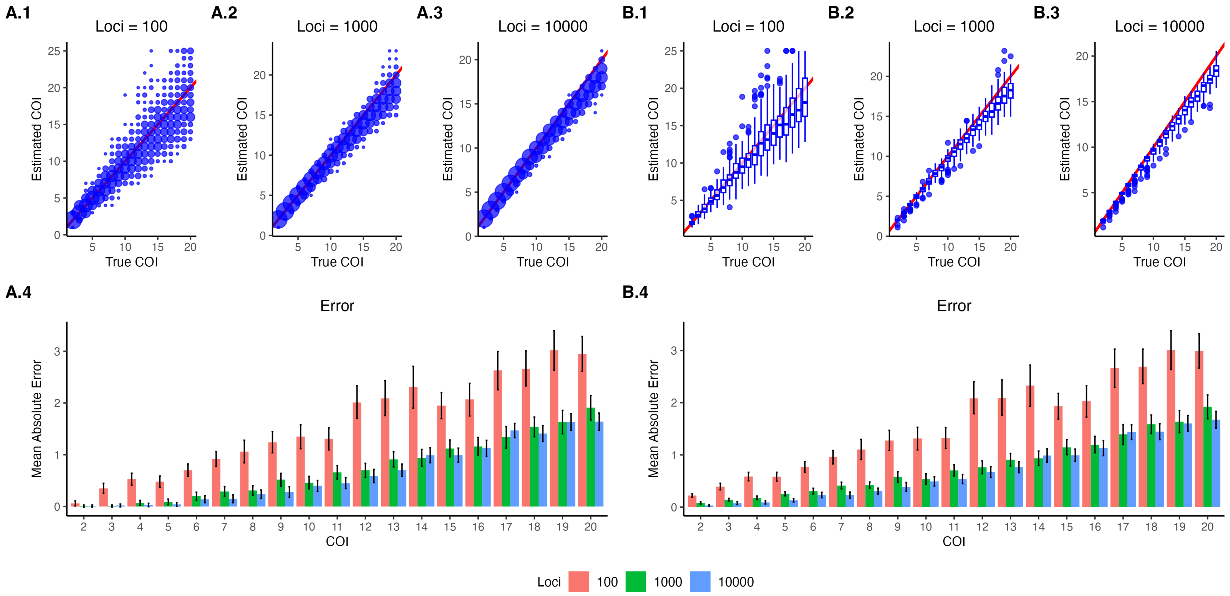

Fig M. Performance of the Variant Method with changing number of loci. The performance of the Variant Method is compared across a COI of 2-20. Data was simulated 100 times with a coverage of 200. No error was introduced into our simulations, and no sequencing error was assumed. In all **A** subplots, the discrete estimation is shown, and in all **B** subplots, the continuous estimation is shown. In both cases, the distribution of estimated COI as the number of loci examined changes is plotted. In **A.1**-**A.3**, dot plots are used where point size indicates density. In **B.1**-**B.3**, a boxplot shows the interquartile range, with whiskers indicating the range and outliers indicated by dots. The mean absolute error is shown in **A.4** and **B.4**, with the 95% confidence interval computed from 1,000 bootstrap repetitions at each COI indicated with error bars.

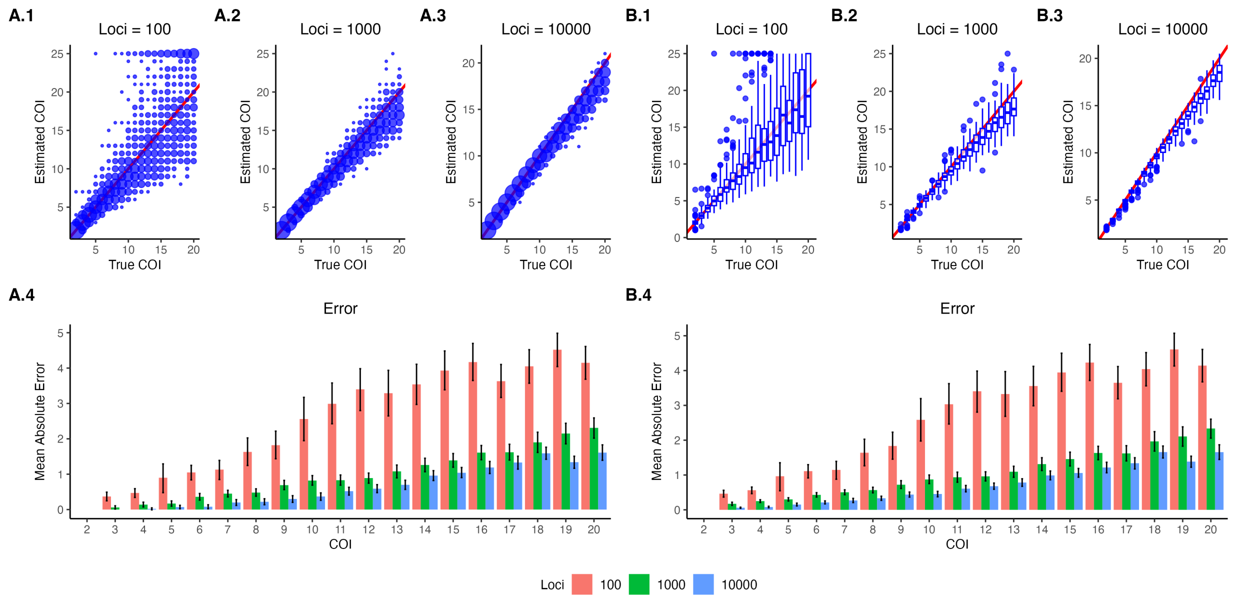

Fig N. Performance of the Frequency Method with changing number of loci. The performance of the Frequency Method is compared across a COI of 2-20. Data was simulated 100 times with a coverage of 200. No error was introduced into our simulations, and no sequencing error was assumed. In all **A** subplots, the discrete estimation is shown, and in all **B** subplots, the continuous estimation is shown. In both cases, the distribution of estimated COI as the number of loci examined changes is plotted. In **A.1**-**A.3**, dot plots are used where point size indicates density. In **B.1**-**B.3**, a boxplot shows the interquartile range, with whiskers indicating the range and outliers indicated by dots. The mean absolute error is shown in **A.4** and **B.4**, with the 95% confidence interval computed from 1,000 bootstrap repetitions at each COI indicated with error bars.

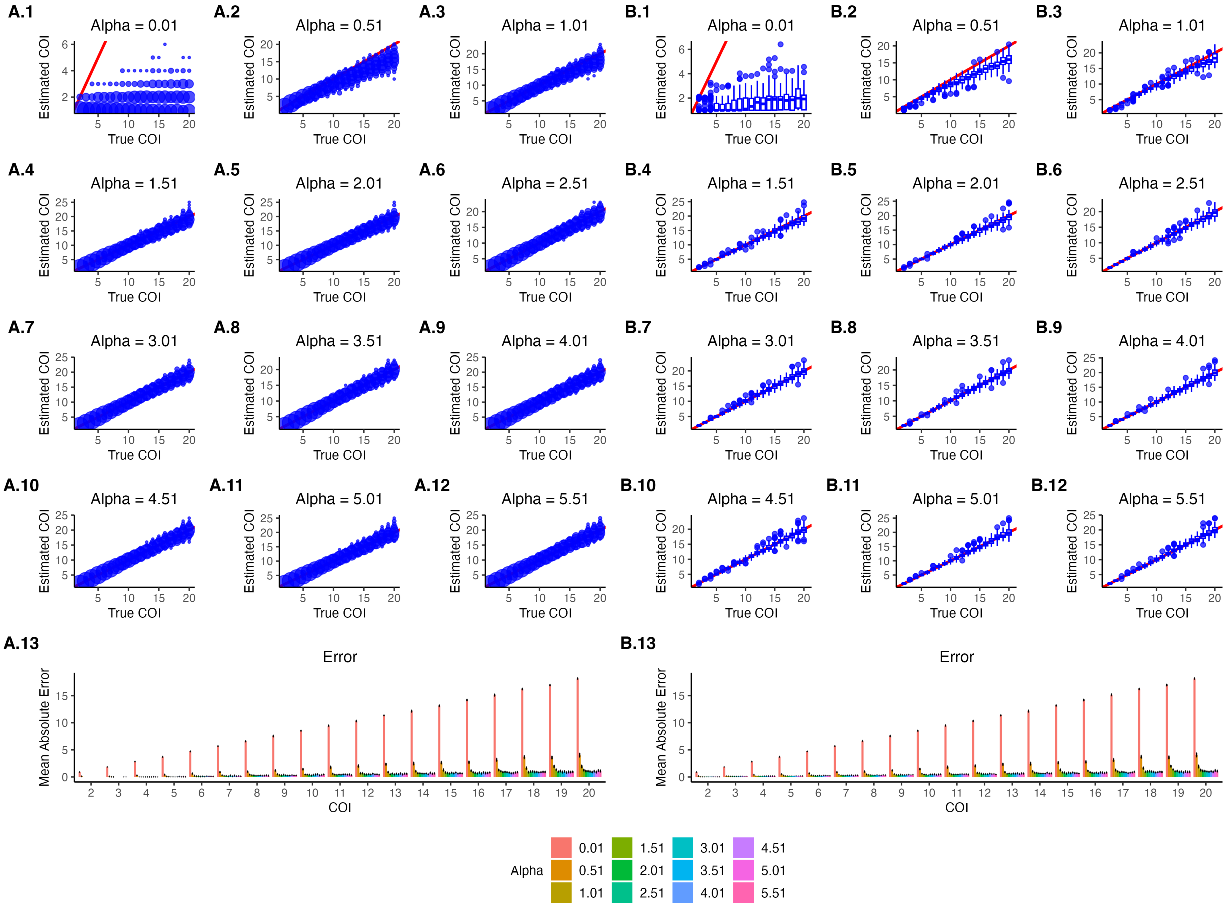

Fig O. Performance of the Variant Method with changing alpha. The performance of the Variant Method is compared across a COI of 2-20. Data was simulated 100 times from 1,000 loci with a coverage of 200. No error was introduced into our simulations, and no sequencing error was assumed. In all **A** subplots, the discrete estimation is shown, and in all **B** subplots, the continuous estimation is shown. In both cases, the distribution of estimated COI as the value of alpha changes is plotted. In **A.1**-**A.12**, dot plots are used where point size indicates density. In **B.1**-**B.12**, a boxplot shows the interquartile range, with whiskers indicating the range and outliers indicated by dots. The mean absolute error is shown in **A.13** and **B.13**, with the 95% confidence interval computed from 1,000 bootstrap repetitions at each COI indicated with error bars.

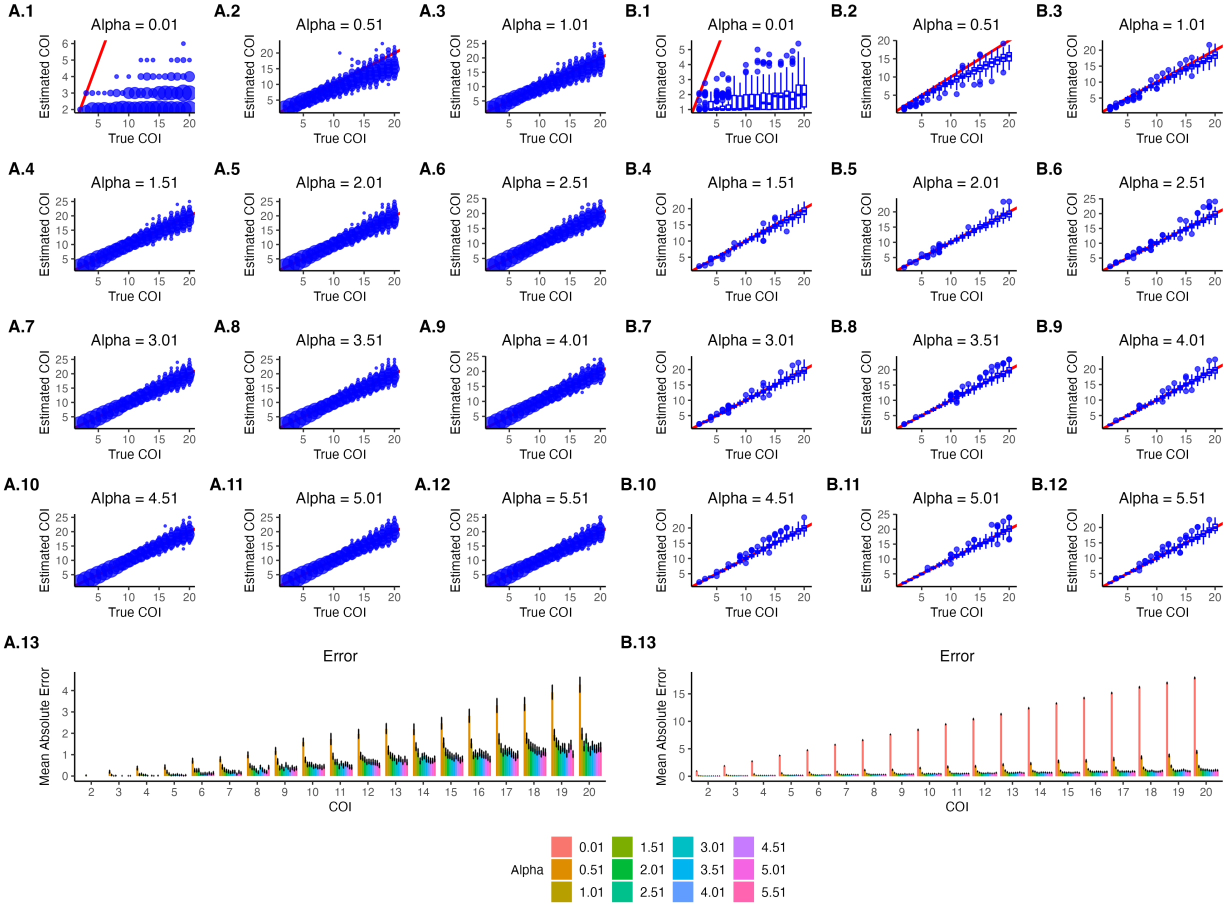

Fig P. Performance of the Frequency Method with changing alpha. The performance of the Frequency Method is compared across a COI of 2-20. Data was simulated 100 times from 1,000 loci with a coverage of 200. No error was introduced into our simulations, and no sequencing error was assumed. In all **A** subplots, the discrete estimation is shown, and in all **B** subplots, the continuous estimation is shown. In both cases, the distribution of estimated COI as the value of alpha changes is plotted. In **A.1**-**A.12**, dot plots are used where point size indicates density. In **B.1**-**B.12**, a boxplot shows the interquartile range, with whiskers indicating the range and outliers indicated by dots. The mean absolute error is shown in **A.13** and **B.13**, with the 95% confidence interval computed from 1,000 bootstrap repetitions at each COI indicated with error bars.

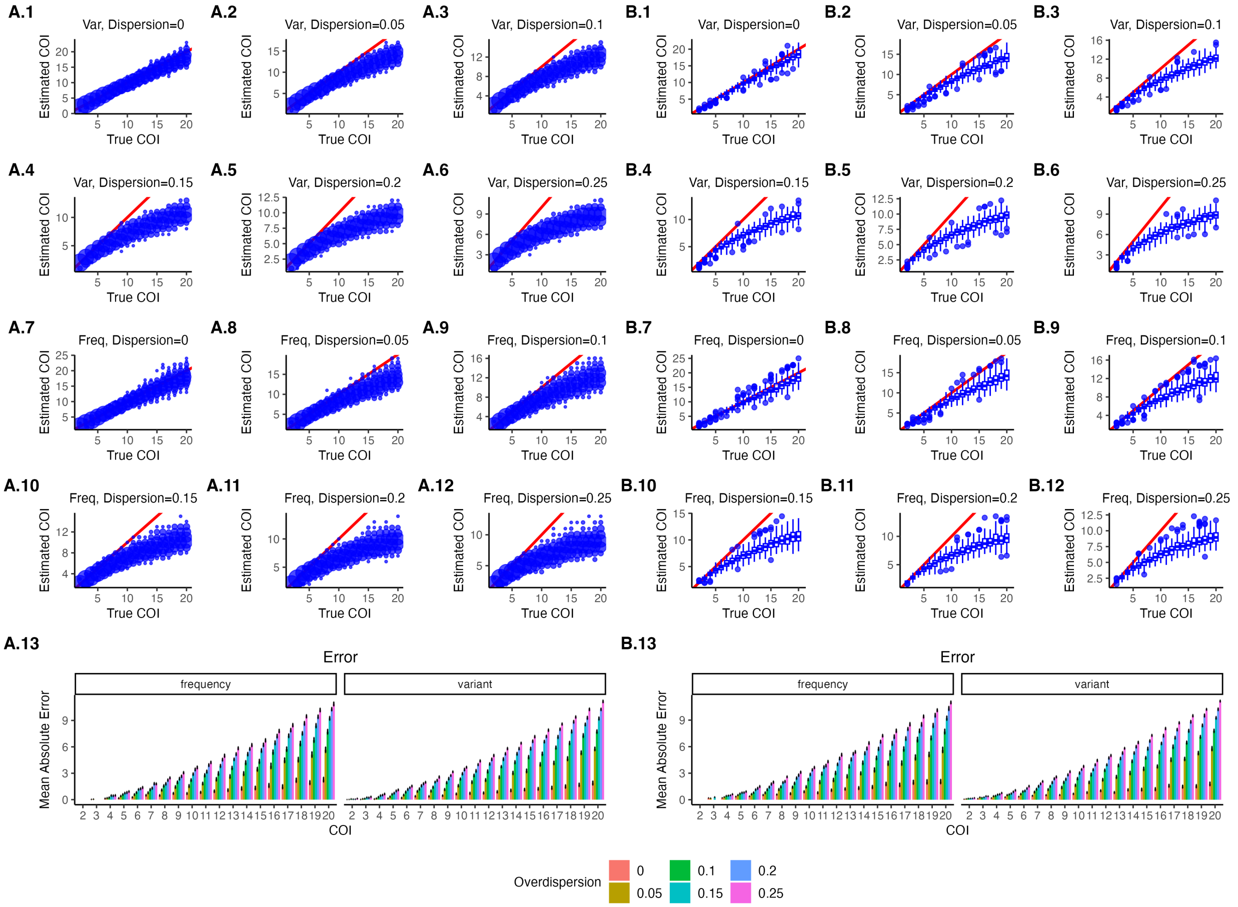

Fig Q. Performance with changing overdispersion. The performance of the Variant Method and the Frequency Method is compared across a COI of 2-20. Data was simulated 100 times from 1,000 loci with a coverage of 200. No error was introduced into our simulations, and no sequencing error was assumed. In all **A** subplots, the discrete estimation is shown, and in all **B** subplots, the continuous estimation is shown. The distribution of estimated COI for the Variant Method (**A.1**-**A.6**, **B.1**-**B.6**) and the Frequency Method (**A.7**-**A.12**, **B.7**-**B.12**) is plotted. In **A.1**-**A.12**, dot plots are used where point size indicates density. In **B.1**-**B.12**, a boxplot shows the interquartile range, with whiskers indicating the range and outliers indicated by dots. The mean absolute error is shown in **A.13** and **B.13**, with the 95% confidence interval computed from 1,000 bootstrap repetitions at each COI indicated with error bars.

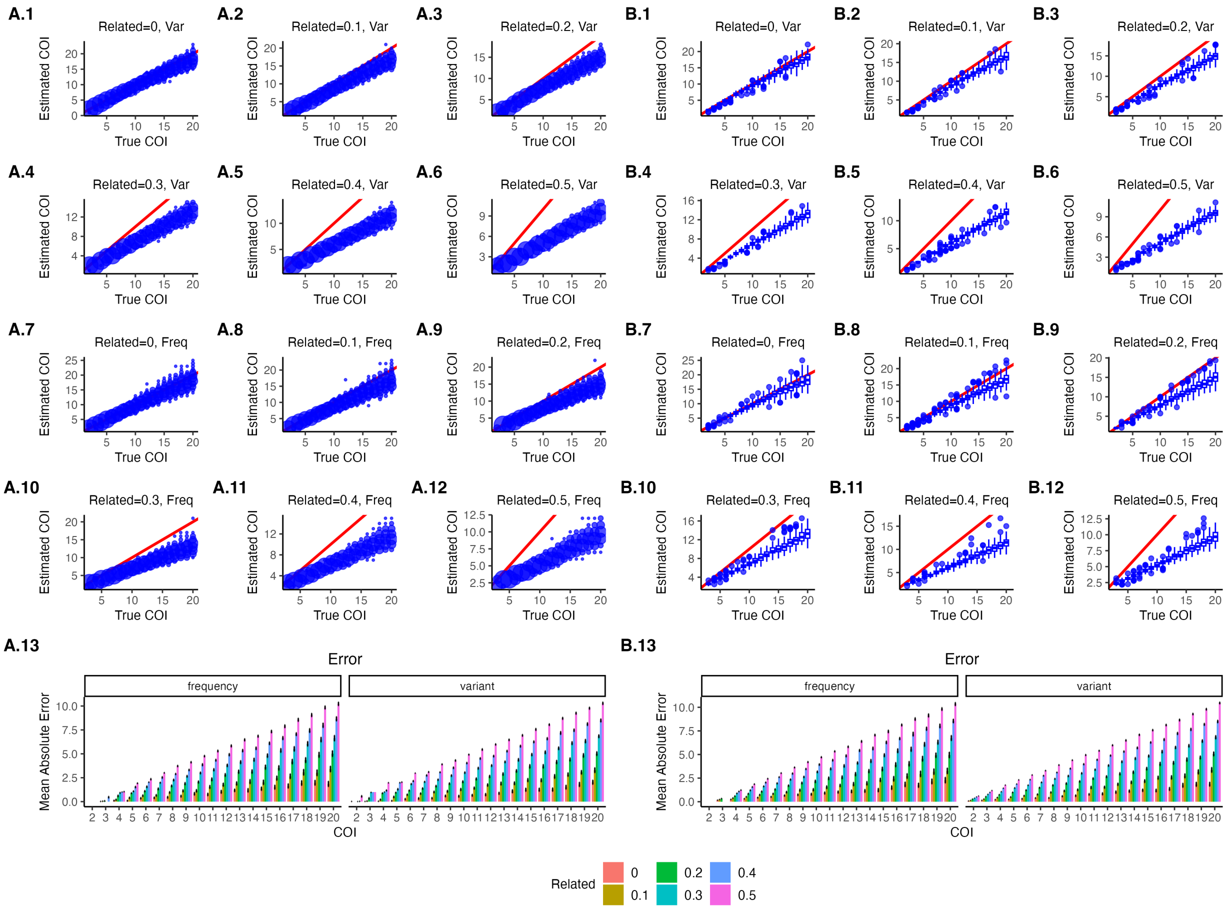

Fig R. Performance with changing relatedness. The performance of the Variant Method and the Frequency Method is compared across a COI of 2-20. Data was simulated 100 times from 1,000 loci with a coverage of 200. No error was introduced into our simulations, and no sequencing error was assumed. In all **A** subplots, the discrete estimation is shown, and in all **B** subplots, the continuous estimation is shown. The distribution of estimated COI for the Variant Method (**A.1**-**A.6**, **B.1**-**B.6**) and the Frequency Method (**A.7**-**A.12**, **B.7**-**B.12**) is plotted. In **A.1**-**A.12**, dot plots are used where point size indicates density. In **B.1**-**B.12**, a boxplot shows the interquartile range, with whiskers indicating the range and outliers indicated by dots. The mean absolute error is shown in **A.13** and **B.13**, with the 95% confidence interval computed from 1,000 bootstrap repetitions at each COI indicated with error bars.

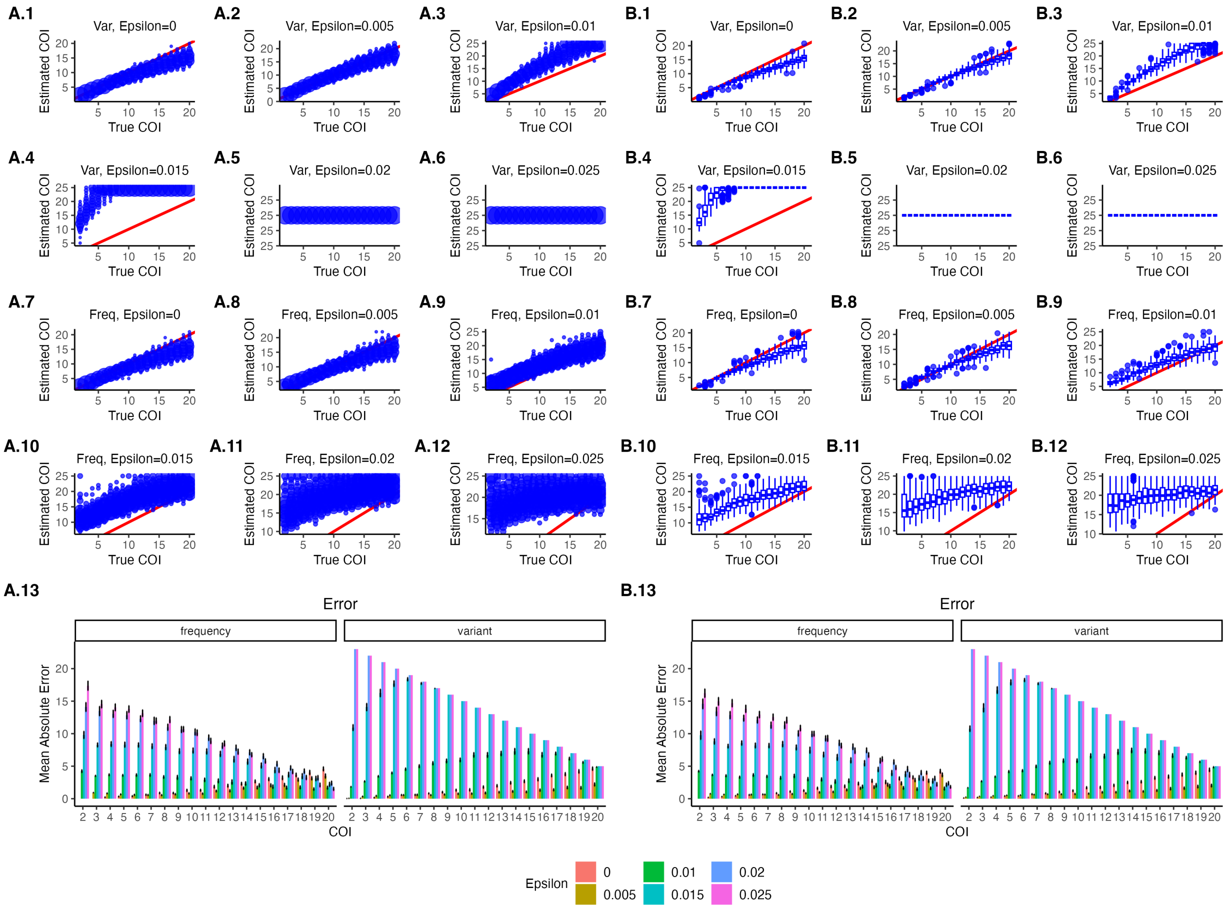

Fig S. Performance with changing epsilon. The performance of the Variant Method and the Frequency Method is compared across a COI of 2-20. Data was simulated 100 times from 1,000 loci with a coverage of 200. A varying error rate, known as epsilon, was introduced into our simulations. This parameter indicates the probability that a single read is miscalled as the other allele. The assumed sequence error, i.e., the error we believe exists in our simulated data, was set to 0.01. In all **A** subplots, the discrete estimation is shown, and in all **B** subplots, the continuous estimation is shown. In both cases, the distribution of estimated COI as epsilon changes is plotted. The distribution of estimated COI for the Variant Method (**A.1-A.6**, **B.1-B.6**) and the Frequency Method (**A.7-A.12**, **B.7-B.12**) is plotted. In **A.1-A.12**, dot plots are used where point size indicates density. In **B.1-B.12**, a boxplot shows the interquartile range, with whiskers indicating the range and outliers indicated by dots. The mean absolute error is shown in **A.13** and **B.13**, with the 95% confidence interval computed from 1,000 bootstrap repetitions at each COI indicated with error bars.

L.1.1 Comparison to *THE REAL McCOIL*

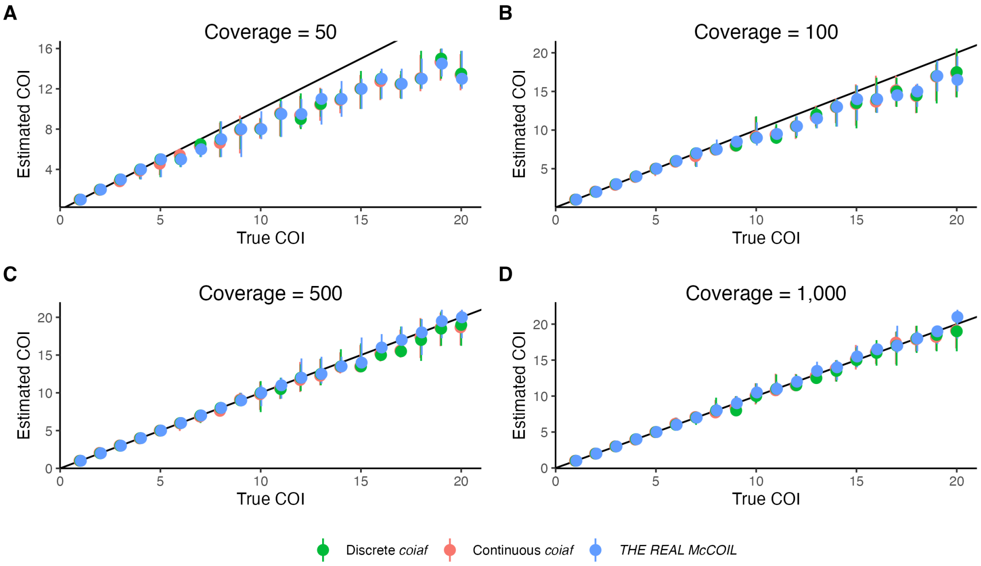

Fig T. Performance with changing coverage. The performance of the discrete and continuous Variant Method is compared to *THE REAL McCOIL* across a COI of 2-20. Data were simulated for each value of the COI 10 times from 1,000 loci. No error was introduced into our simulations, and no sequencing error was assumed. The subplots show the estimation methods' performance as the coverage varies. In all subplots, the median value of the COI was plotted for each estimation method. The vertical bars indicate the 95% quantile range. The color of each point and its corresponding error bar corresponds to each of the three estimation methods.

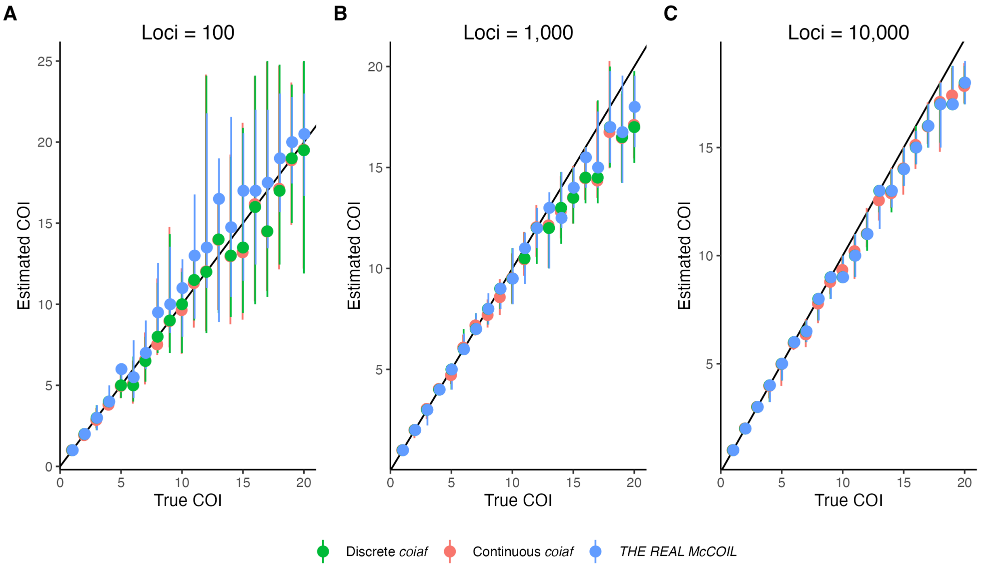

Fig U. Performance with changing loci. The performance of the discrete and continuous Variant Method is compared to *THE REAL McCOIL* across a COI of 2-20. Data were simulated for each value of the COI 10 times with a coverage of 200. No error was introduced into our simulations, and no sequencing error was assumed. The subplots show the estimation methods' performance as the number of loci varies. In all subplots, the median value of the COI was plotted for each estimation method. The vertical bars indicate the 95% quantile range. The color of each point and its corresponding error bar corresponds to each of the three estimation methods.

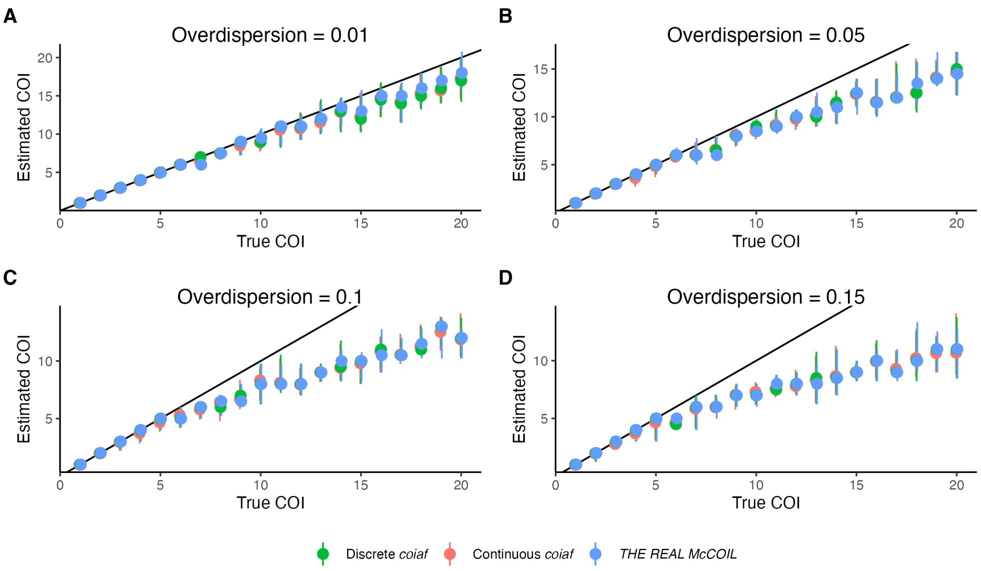

Fig V. Performance with changing overdispersion. The performance of the discrete and continuous Variant Method is compared to *THE REAL McCOIL* across a COI of 2-20. Data were simulated for each value of the COI 10 times from 1,000 loci with a coverage of 200. No error was introduced into our simulations, and no sequencing error was assumed. The subplots show the estimation methods' performance as the overdispersion parameter varies. In all subplots, the median value of the COI was plotted for each estimation method. The vertical bars indicate the 95% quantile range. The color of each point and its corresponding error bar corresponds to each of the three estimation methods.

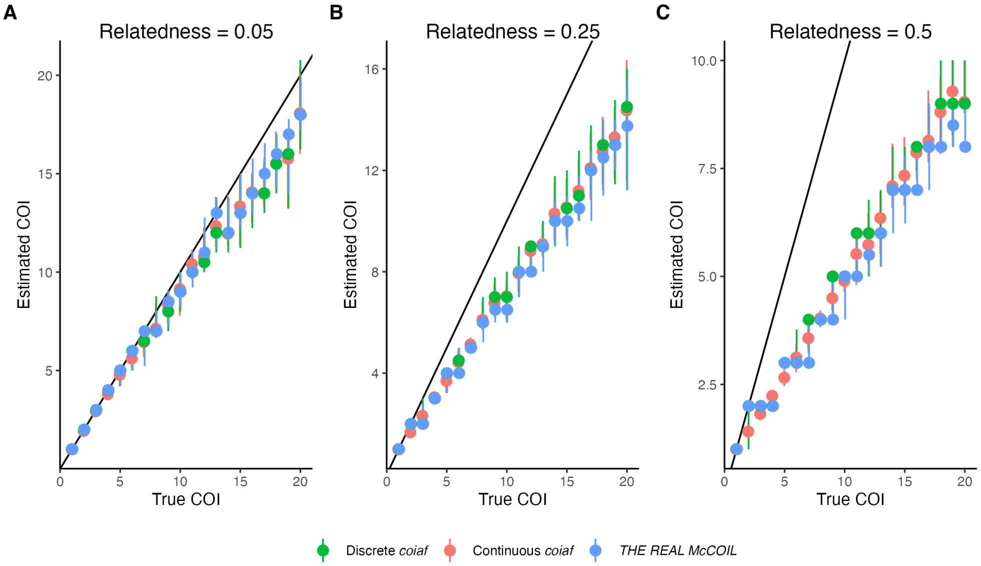

Fig W. Performance with changing relatedness. The performance of the discrete and continuous Variant Method is compared to *THE REAL McCOIL* across a COI of 2-20. Data were simulated for each value of the COI 10 times from 1,000 loci with a coverage of 200. No error was introduced into our simulations, and no sequencing error was assumed. The subplots show the estimation methods' performance as the level of relatedness varies. In all subplots, the median value of the COI was plotted for each estimation method. The vertical bars indicate the 95% quantile range. The color of each point and its corresponding error bar corresponds to each of the three estimation methods.

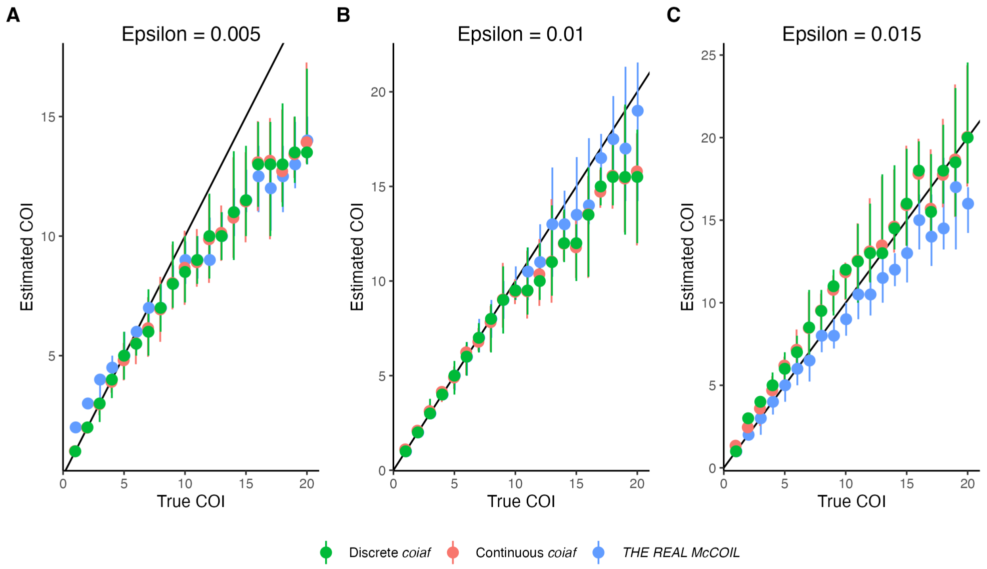

Fig X. Performance with changing epsilon. The performance of the discrete and continuous Variant Method is compared to *THE REAL McCOIL* across a COI of 2-20. Data were simulated for each value of the COI 10 times from 1,000 loci with a coverage of 200. The subplots show the estimation methods' performance as the amount of sequence error introduced into the simulations, known as epsilon, varies. For each subplot, both *THE REAL McCOIL* and *coiaf* estimated the sequence error across the population independently. This can explain the change in bias between subplots **A** and **B** and subplot **C**. In all subplots, the median value of the COI was plotted for each estimation method. The vertical bars indicate the 95% quantile range. The color of each point and its corresponding error bar corresponds to each of the three estimation methods.

L.1.2 Estimated PLMAF

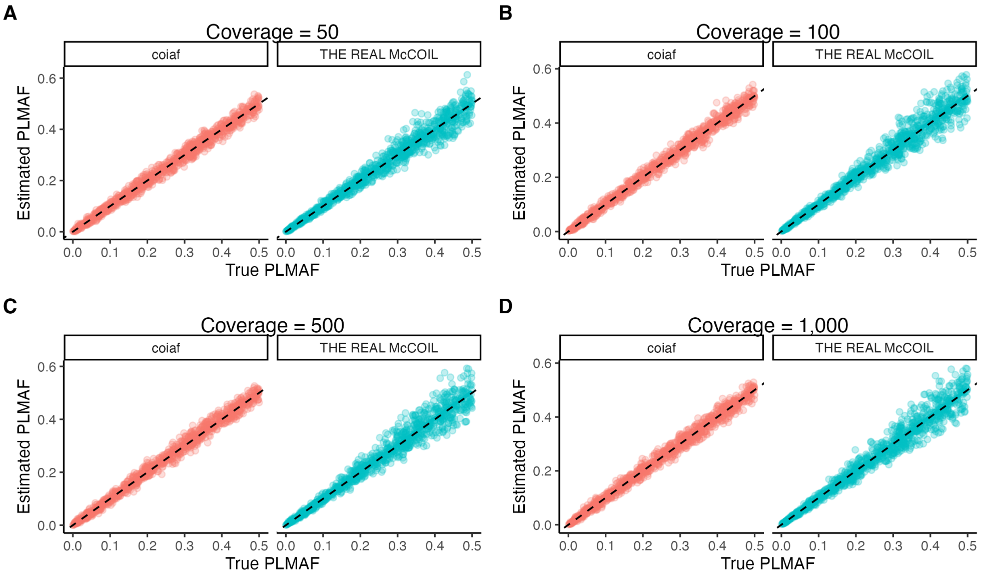

Fig Y. PLMAF with changing coverage. Data were simulated 10 times across a range of COIs from 2-20 with 1,000 loci. The true estimated population-level minor allele frequency (PLMAF), i.e., the PLMAF that data was simulated with, was compared to the PLMAF estimated by *coiaf* and *THE REAL McCOIL*. Each subplot illustrates this comparison for data simulated with a different read depth coverage. The dashed black line in each subplot represents the line $y = x$.

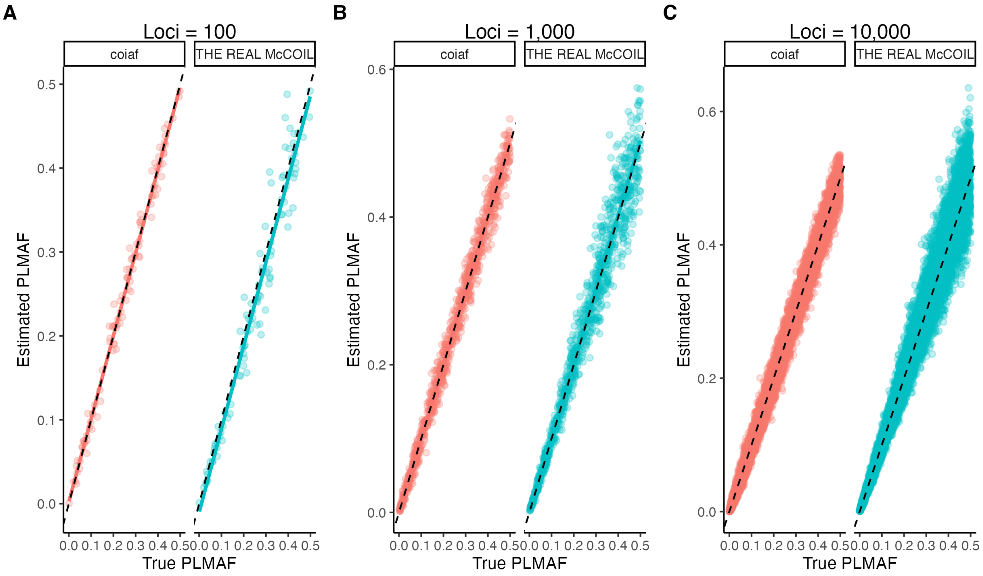

Fig Z. PLMAF with changing loci. Data were simulated 10 times across a range of COIs from 2-20 with a coverage of 200. The true estimated population-level minor allele frequency (PLMAF), i.e., the PLMAF that data was simulated with, was compared to the PLMAF estimated by *coiaf* and *THE REAL McCOIL*. Each subplot illustrates this comparison for data simulated with a different number of loci. The dashed black line in each subplot represents the line $y = x$.

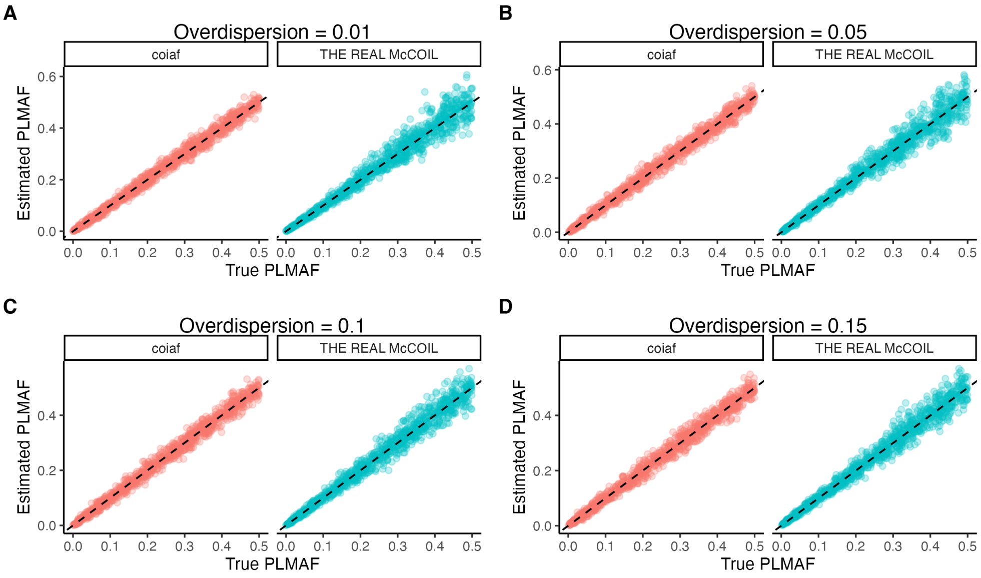

Fig AA. PLMAF with changing overdispersion. Data were simulated 10 times across a range of COIs from 2-20 with 1,000 loci and a coverage of 200. The true estimated population-level minor allele frequency (PLMAF), i.e., the PLMAF that data was simulated with, was compared to the PLMAF estimated by *coiaf* and *THE REAL McCOIL*. Each subplot illustrates this comparison for data simulated with a different level of overdispersion. The dashed black line in each subplot represents the line $y = x$.

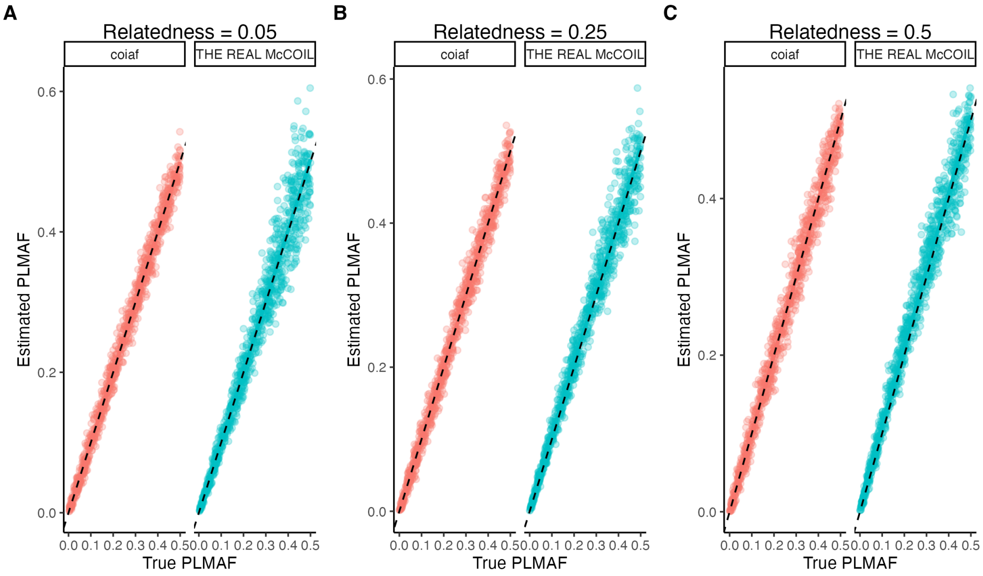

Fig AB. PLMAF with changing relatedness. Data were simulated 10 times across a range of COIs from 2-20 with 1,000 loci and a coverage of 200. The true estimated population-level minor allele frequency (PLMAF), i.e., the PLMAF that data was simulated with, was compared to the PLMAF estimated by *coiaf* and *THE REAL McCOIL*. Each subplot illustrates this comparison for data simulated with a different level of relatedness. The dashed black line in each subplot represents the line $y = x$.

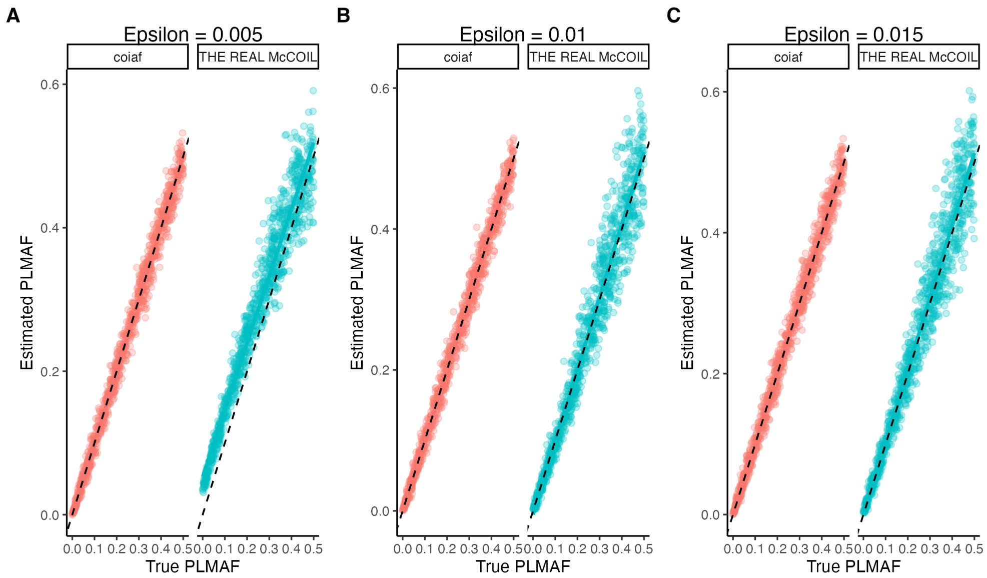

Fig AC. PLMAF with changing epsilon. Data were simulated 10 times across a range of COIs from 2-20 with 1,000 loci and a coverage of 200. The true estimated population-level minor allele frequency (PLMAF), i.e., the PLMAF that data was simulated with, was compared to the PLMAF estimated by *coiaf* and *THE REAL McCOIL*. Each subplot illustrates this comparison for data simulated with different amounts of sequence error introduced into the simulations, known as epsilon. The dashed black line in each subplot represents the line $y = x$.

L.2 Clustering real data samples

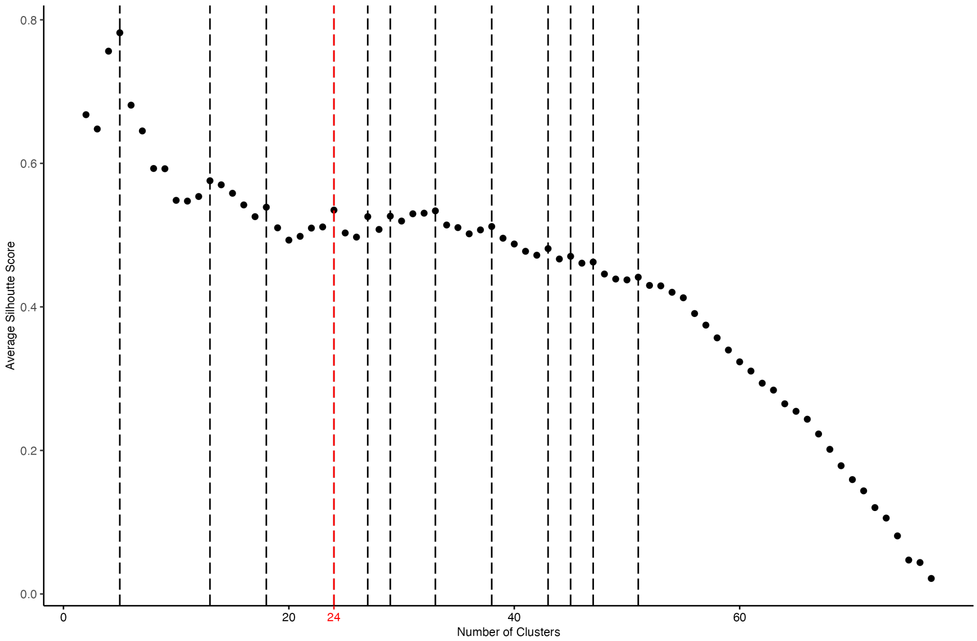

Fig AD. Silhouette plot. The x-axis represents the number of clusters the data was split into. The y-axis indicates the average silhouette score, a measure of cluster validity and strength. The silhouette score can provide a measure to select the optimal number of clusters [9].

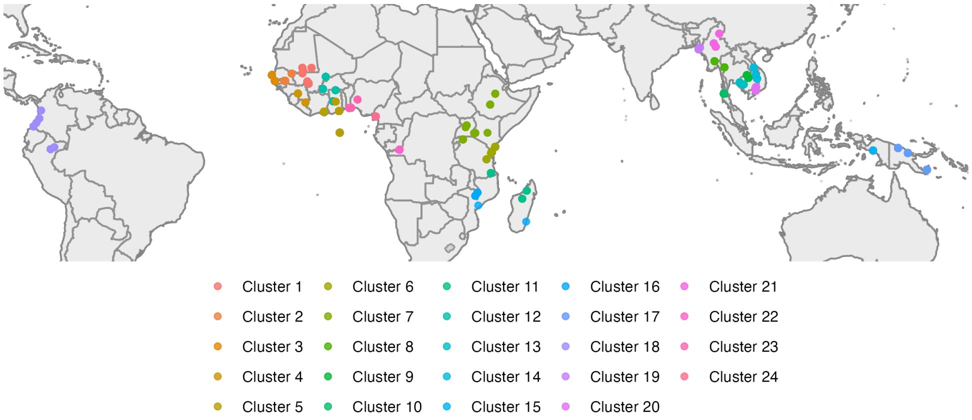

Fig AE. Location of 24 clusters. A plot of each location data was sampled from. The color of the point indicates which of the 24 clusters data was assigned to. Map data was obtained from Natural Earth (medium scale data, 1:50m), which is in the public domain.

L.3 Real data

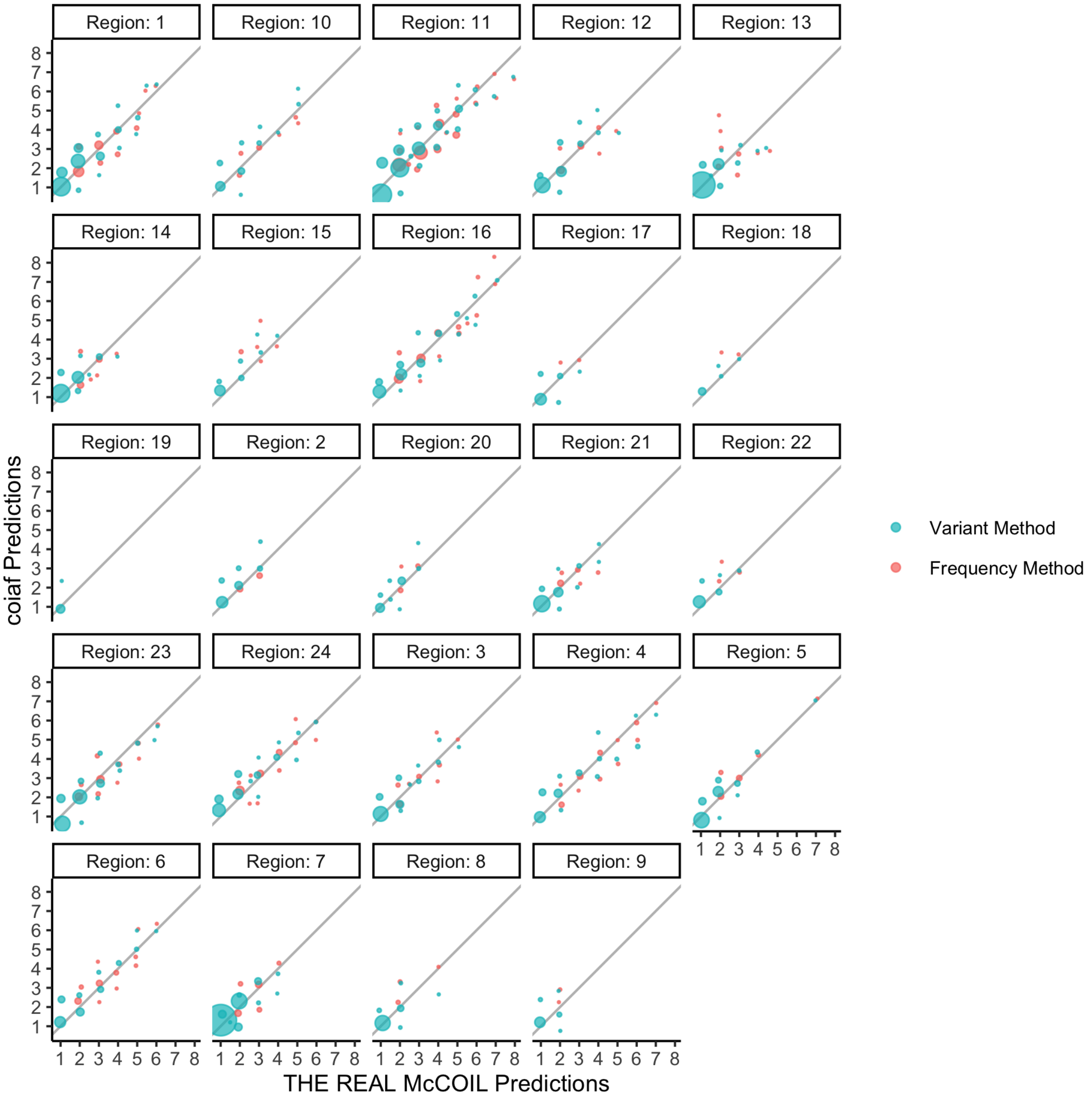

Fig AF. Comparison between *THE REAL McCOIL* and discrete *coiaf* across all regions. For each of the 24 regions, we plot the discrete estimate of the COI for our methods compared to the results of *THE REAL McCOIL*. The estimated COI using *THE REAL McCOIL* is plotted on the x-axis. For all samples of a specific COI value, we plot the results of our methods on the y-axis. If our methods and *THE REAL McCOIL* predict the same COI, we expect that data points will fall along the line $y = x$.

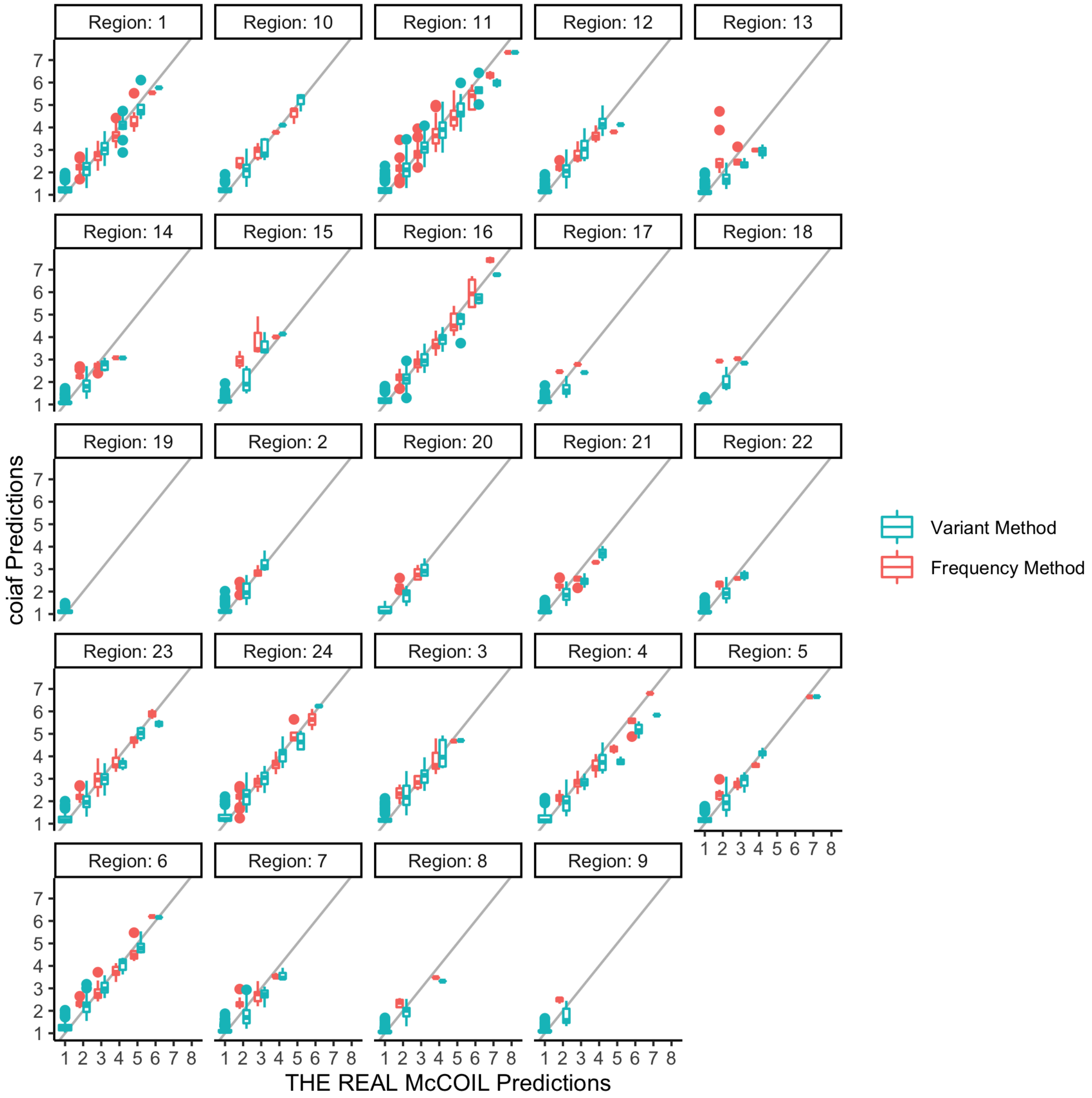

Fig AG. Comparison between *THE REAL McCOIL* and continuous *coiaf* across all regions. For each of the 24 regions, we plot the continuous estimate of the COI for our methods compared to the results of *THE REAL McCOIL*. The estimated COI using *THE REAL McCOIL* is plotted on the x-axis. For all samples of a specific COI value, a boxplot shows the interquartile range, with whiskers indicating the range and outliers indicated with dots of the results of our methods on the y-axis. If our methods and *THE REAL McCOIL* predict the same COI, we expect that data points will fall along the line $y = x$.

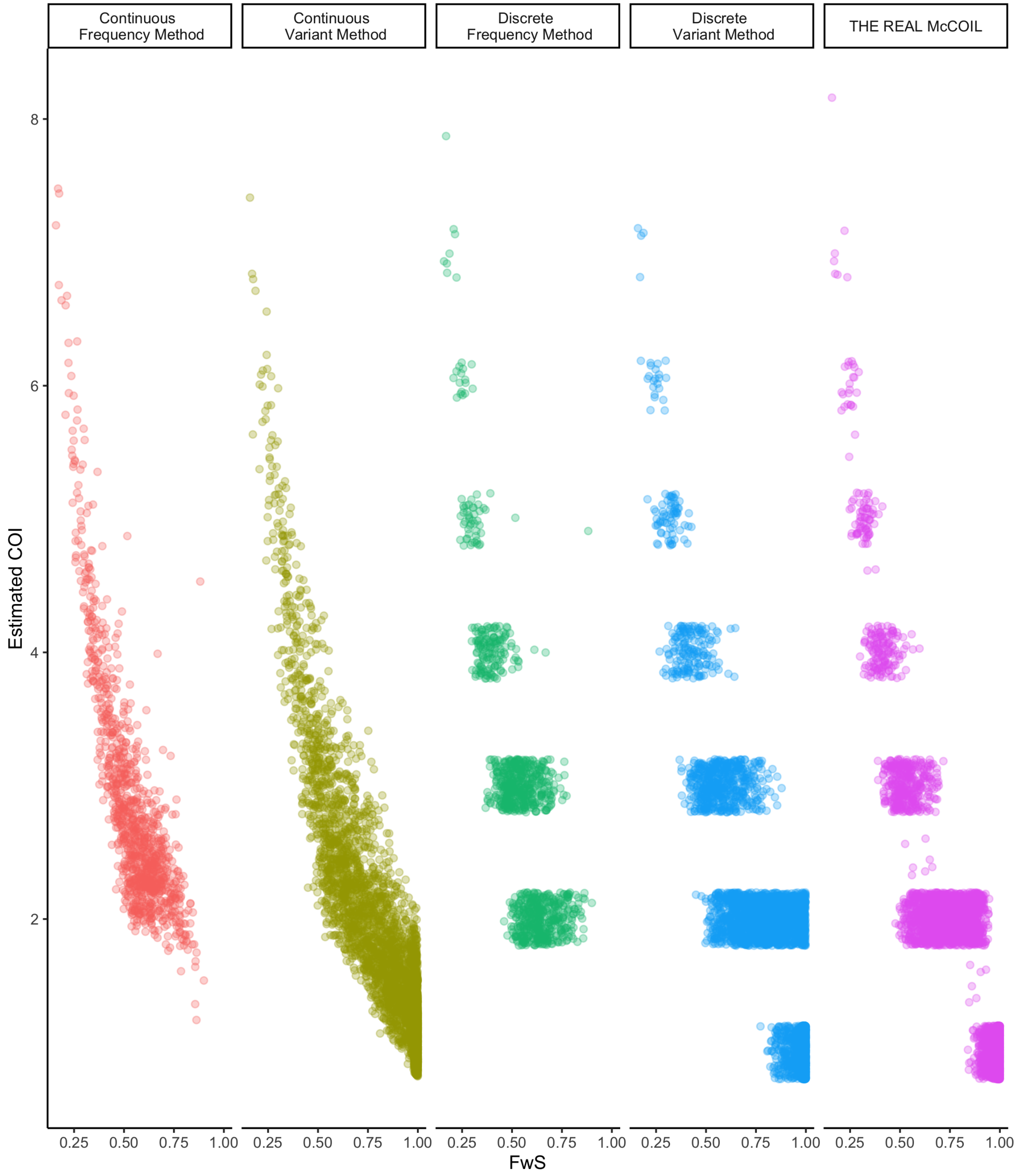

Fig AH. Comparison between estimation methods and *FwS*. We compare the estimated COI for all samples to the measured *FwS* metric, a measure of the within-host genetic diversity, where a lower *FwS* indicates a higher within-host diversity. The x-axis indicates the *FwS*. The y-axis indicates the estimated COI. We compare various methods using *coiaf* and *THE REAL McCOIL*. Note that each band of data in the Discrete Frequency Method, the Discrete Variant Method, and *THE REAL McCOIL* corresponds to the integer value of the COI—noise in the y-direction has been added for interpretability.

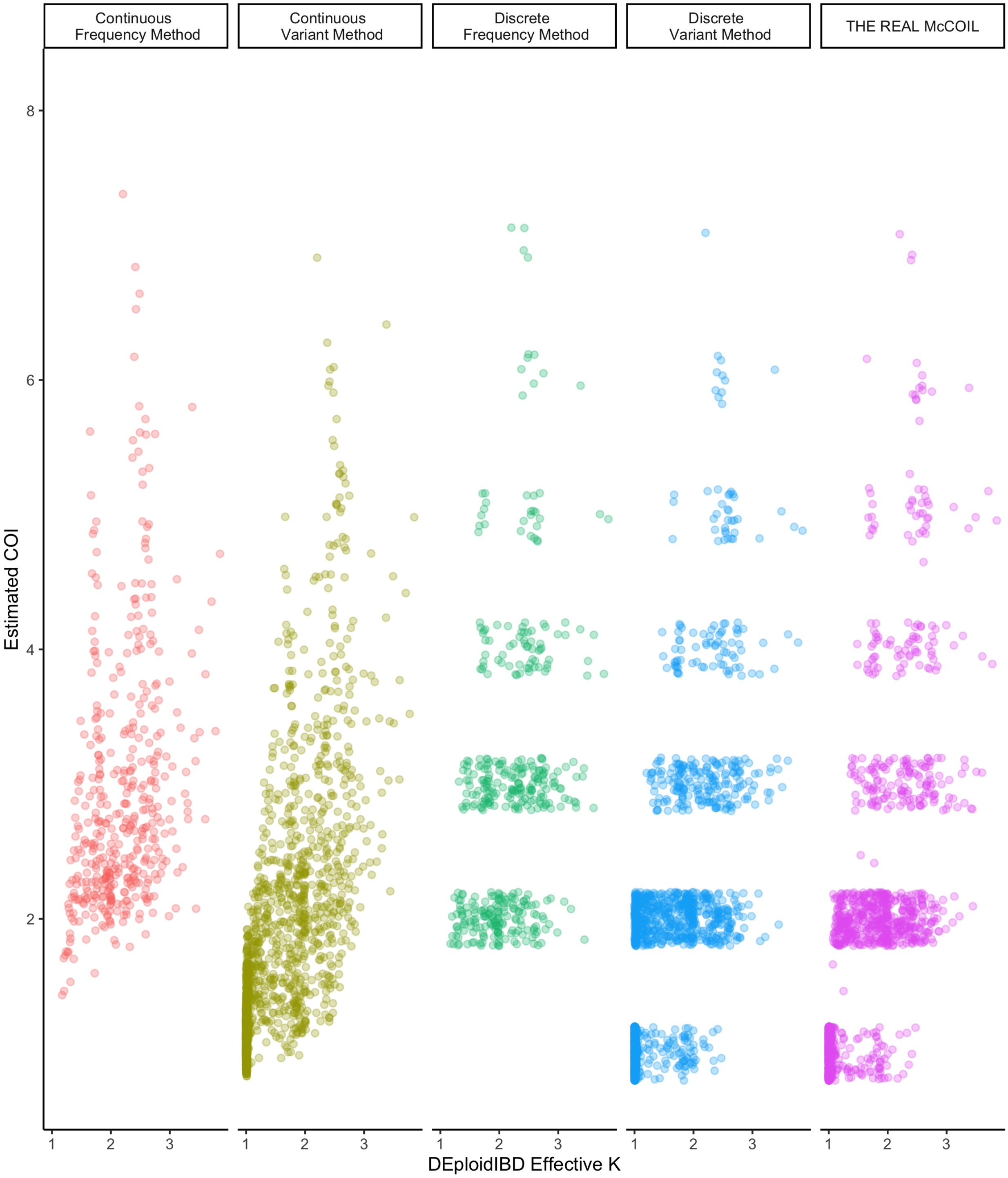

Fig AI. Comparison between estimation methods and *DEploidIBD* effective K_e. We compare the estimated COI for all samples to the measured *DEploidIBD* effective K_e metric, a measure of the complexity of infection and relative parasite density of each strain in that mixed infection, such that $K_e = 1/\sum(w_i^2)$, where w_i is the proportion of the ith strain [10]. The x-axis indicates the effective K_e. The y-axis indicates the estimated COI. We compare various methods using *coiaf* and *THE REAL McCOIL*. Note that each band of data in the Discrete Frequency Method, the Discrete Variant Method, and *THE REAL McCOIL* corresponds to the integer value of the COI—noise in the y-direction has been added for interpretability.

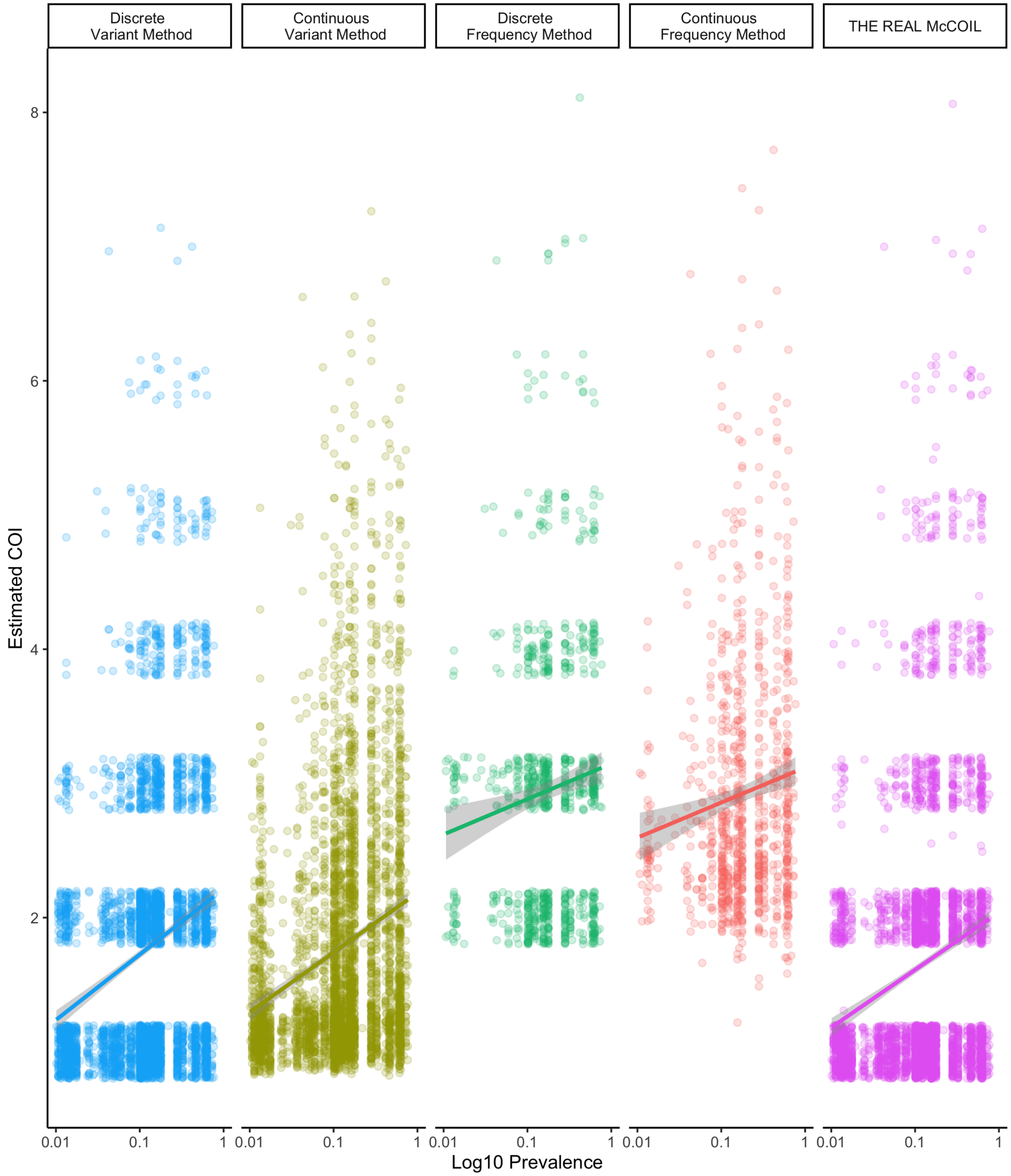

Fig AJ. Comparison between estimation methods and malaria prevalence. We examine the relationship between COI and malaria prevalence. The x-axis indicates the prevalence of malaria, which was obtained from the Malaria Atlas Project [11–13]. The y-axis indicates the estimated COI. We compare various methods using *coiaf* and *THE REAL McCOIL*. Note that each band of data in the Discrete Variant Method, the Discrete Frequency Method, and *THE REAL McCOIL* corresponds to the integer value of the COI—noise in the y-direction has been added for interpretability.

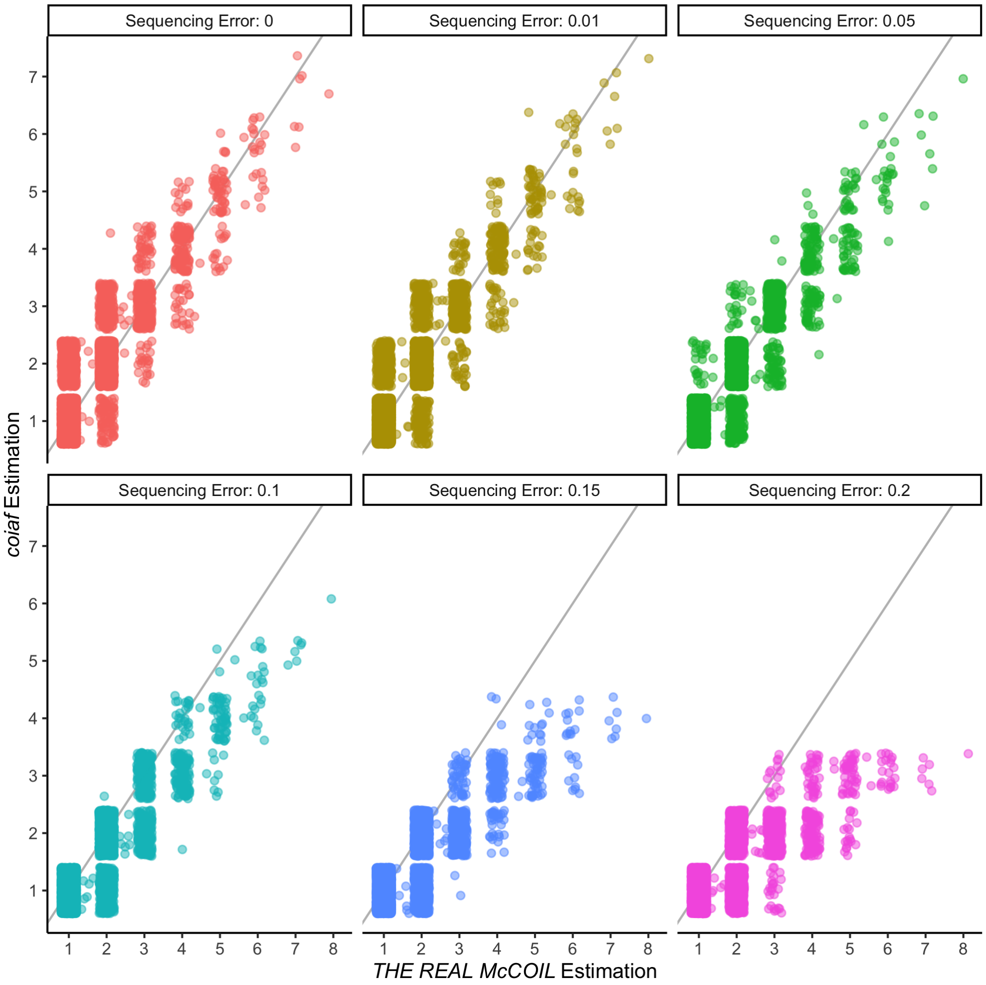

Fig AK. Varying assumed sequence error. To appreciate how our estimates of the COI are impacted by sequencing error, we plot estimates when varying levels of sequence error are assumed in the population. The level of error assumed and accounted for in our estimations is denoted by the color of each point and noted above each plot. Our estimates are plotted against the estimates of *THE REAL McCOIL*, where we let the algorithm infer the level of sequencing error in the population. For all samples of a specific COI value, we plot the results of the discrete Variant Method on the y-axis for varying levels of error.

References

1. Paul SR. A three-parameter generalization of the binomial distribution. Communications in Statistics - Theory and Methods. 1985;14(6):1497–1506. doi:10.1080/03610928508828990.

2. Canty A, Ripley BD. boot: Bootstrap r (s-plus) functions; 2021.

3. Hester J. bench: High Precision Timing of R Expressions; 2020. Available from: `https://CRAN.R-project.org/package=bench`.

4. Clarke E, Sherrill-Mix S. ggbeeswarm: Categorical scatter (violin point) plots; 2017. Available from: `https://CRAN.R-project.org/package=ggbeeswarm`.

5. McDonald JH. Handbook of Biological Statistics. 3rd ed. Sparky House Publishing, Baltimore, Maryland; 2014.

6. Gelman A, Rubin DB. Markov chain Monte Carlo methods in biostatistics. Statistical Methods in Medical Research. 1996;5(4):339–355. doi:10.1177/096228029600500402.

7. Chang HH, Worby CJ, Yeka A, Nankabirwa J, Kamya MR, Staedke SG, et al. THE REAL McCOIL: A method for the concurrent estimation of the complexity of infection and SNP allele frequency for malaria parasites. PLOS Computational Biology. 2017;13(1):e1005348. doi:10.1371/journal.pcbi.1005348.

8. Barbujani G. Autocorrelation of Gene Frequencies under Isolation by Distance. Genetics. 1987;117(4):777–782.

9. Rousseeuw PJ. Silhouettes: A graphical aid to the interpretation and validation of cluster analysis. Journal of Computational and Applied Mathematics. 1987;20:53–65. doi:10.1016/0377-0427(87)90125-7.

10. Zhu SJ, Hendry JA, Almagro-Garcia J, Pearson RD, Amato R, Miles A, et al. The origins and relatedness structure of mixed infections vary with local prevalence of P. falciparum malaria. eLife. 2019;8:e40845. doi:10.7554/eLife.40845.

11. Hay SI, Snow RW. The Malaria Atlas Project: Developing Global Maps of Malaria Risk. PLOS Medicine. 2006;3(12):e473. doi:10.1371/journal.pmed.0030473.

12. Guerra CA, Hay SI, Lucioparedes LS, Gikandi PW, Tatem AJ, Noor AM, et al. Assembling a global database of malaria parasite prevalence for the Malaria Atlas Project. Malaria Journal. 2007;6(1):17. doi:10.1186/1475-2875-6-17.

13. Pfeffer D, Lucas T, May D, Harris J, Rozier J, Twohig K, et al. malariaAtlas: an R interface to global malariometric data hosted by the Malaria Atlas Project. Malaria Journal. 2018;17(1):352. doi:10.1186/s12936-018-2500-5.
