# Supplementary material for: coiaf: Directly estimating complexity of infection with allele frequencies
Source: PLoS Comput Biol. 2023 Jun 9;19(6):e1010247. doi: 10.1371/journal.pcbi.1010247 (PMC10310041; doi:10.1371/journal.pcbi.1010247)
Supplement: Attachment — Submitted filename: Response to Reviewers.pdf [file pcbi.1010247.s003.pdf]

Dear Reviewers,

We would like to greatly express our thanks for taking the time to review our manuscript so thoroughly. Your valuable feedback and insightful comments have greatly improved the quality of our work. We are very grateful for the time and effort taken in helping us to improve our manuscript.

In particular, we would like to thank you for raising important points that required further elaboration and clarification about our methods and parameters in the sensitivity analysis. In response, we have added additional results, discussions, appendices, and supplementary figures to better articulate our approach.

We also would like to apologize for the delay in our response to your comments. We have been conducting numerous additional simulations, both using our own methods and THE REAL McCOIL, in order to address your concerns and provide a thorough response to your comments requesting a direct head-to-head comparison of the two methods against the same simulated data. We hope that our revised manuscript meets your expectations and effectively communicates the significance of our research.

Reviewer #1

Major Comments

1. *Formalisation of the objective functions: Usually in weighted least squares, the weighted sum of squared residuals is minimised (e.g. see page 389 of Gelman and Hill, Data analysis using regression and multilevel/hierarchical models), not the sum of weighted residuals squared (coiaf). As such, the COI estimate of coiaf is not the MLE of a weighted regression, but something else which I don't quite understand. This irregular approach might explain the non-linear relationship between coverage and performance (line 212) and the poor coverage of confidence intervals (Appendix E and plots A.3. and B.3 of Figs 4 and 5 of Appendix K), which I'm also struggling to understand. It would make more sense to do regular weighted least squares. At the very least, the authors should 1) verify numerically that this alternative estimator is consistent, i.e., for a fixed large COI, 20 say, show that as the number of data points increases (x axis) the estimates (y axis) converge to the true COI; and 2) include a caveat highlighting that the approach is irregular*

Response: Thank you for catching this error in our equations in the main text. We minimize the weighted sum of square residuals in our software package.* However, we made a mistake in equations 4-7. We have now corrected our equations to correctly show that the weighted sum of squared residuals is being used. We have also added additional text around the equations to clarify this, stating that we are solving a weighted least squares minimization problem.

* See the following links for the code in which we define the minimization problem: https://github.com/bailey-lab/coiaf/blob/37c87ebddf49c15bb7d99ba41494b3a0305dc477/R/optimize.R#L61-L63 and https://github.com/bailey-lab/coiaf/blob/37c87ebddf49c15bb7d99ba41494b3a0305dc477/R/compute_coi.R#L300-L302.

2. *Model assumptions: The assumptions of the underlying model (see summary above) could be better articulated in the main text (e.g in Materials and methods and again on line 355). Indeed, their implicit nature appears to be misleading:*
    a. *Appendix J details how SNPs in LD are unsuitable for THE REAL McCOIL; however, the same is true of coiaf, given assumption 1.*

Response: Thank you for your comment on the independence assumptions, which we agree we needed to clarify. We have explained these assumptions in the Methods at the end of the Variant Method section and detailed how they impact our methods if violated. We describe how these impact our methods below, which has also been added to the Appendix C.2, Impact of correlation among loci.

THE REAL McCOIL assumes independence, as does the earlier model, *COIL*, on which it is based. This is because dependence between loci, i.e., linkage disequilibrium (LD), violates the assumption that each locus is solely dependent on allele frequencies and can thus be described with a Bernoulli process. However, if sites are not independent, this does not bias the estimator but increases the variance in estimated COI. For example, if two loci are correlated due to LD, and if one locus returns a polymorphic call, the other locus most likely will also be polymorphic. The opposite, however, is also true that if one locus is homozygous, then the other locus likely will be too. Consequently, with very large numbers of loci, these antagonistic effects will cancel each other out. To adhere to the stated assumptions of *THE REAL McCOIL,* however, we used sites that were not in LD (also because if we used all/more polymorphic sites, we would not have been able to run *THE REAL McCOIL* due to the much larger memory requirements).

The variant method of *coiaf* will have the same impact on its variance in COI estimated, as it relies on the same Bernoulli assumptions. However, given the much lower computational overhead of *coiaf,* we can leverage significantly more loci, which will largely offset this variance. The frequency method, which focuses on within sample minor allele frequencies, will be unaffected by correlation among sites.

b. *Under a model that captures the finite nature of the genome, the reduction in variance with increasing loci count (line 221) would asymptote.*

Response: We agree with this feature of the results and have added a sentence about this to the Sensitivity Analysis section of the Results. We state on lines 236-237: "This reduction in variance will eventually asymptote as the number of loci included tends towards the total number of variant sites in the genome." Thank you for this observation.

3. *Misspecification: how does coiaf perform when the two model assumptions (independent loci and unrelated strains) are violated? For a regular MLE, the first is likely to impact CI coverage but not the point estimates, since correlation among SNPs simply reduces the information content in the data.*

Response: Thank you for your interest in this. We have added relatedness to our simulator, which is described in the Appendix G, Simulator Details. In overview, in mixed infections, we simulate the genotype for the first strain as normal by sampling from the population level minor allele frequency. For each following strain, the allele for each locus is either drawn from the population level minor allele frequency or sampled from one of the other strains in the mixed infection, with the probability that we sample from one of the other strains equal to the relatedness.

The impact of relatedness has now been added as an additional sensitivity analysis explored, which shows how increasing relatedness reduces the accuracy of our methods, with increasing relatedness resulting in systematic underestimation of COI (see Appendix L.1, Figure 9). We have also assessed the impact of relatedness in our additional analyses in which we make a head-to-head comparison of *THE REAL McCOIL* against our methods, which both show similar

effects of relatedness (see Appendix L.1.1, Figure 4). In particular, as the relatedness between parasite strains increased, our estimation methods began to underpredict the true COI. However, with 10% relatedness between strains in mixed infections, our methods could still estimate the COI of up to 10 with a low mean absolute error of less than 1. Under prediction of COI increased consistently with increasing relatedness, with 50% relatedness resulting in COI being similarly underestimated by 50%

Regarding independence amongst loci, we have added an explanation of how this impacts our methods in Appendix C.2, Impact of correlation among loci, which also links to our response to your comment #2.

4. *Benchmarking: I am confused about which data were used to benchmark coiaf and THE REAL McCOIL (sorry if I've missed something). The main text mainly implies COI estimates from the pf samples with unknown true COIs were compared, but reference to the "true COI" (line 240) suggests otherwise. Were pf data engineered in silico to generate complex pf infections with known COI? Fig 5 of Appendix K.3 implies some bench marking was done using simulated data. In any case, I expected to see plots that compare error and CI coverage across the two methods.*

Response: Thank you for raising the need here for more clarity on benchmarking. In the results section Comparison to "state-of-the-art" methods, the "true COI" we are assuming is *THE REAL McCOIL,* i.e., it is currently the best method for estimating COI, and here we are comparing the methods against the Pf6k database. In the Sensitivity Analysis section, the true COI is the simulated, known COI. We appreciate this was confusing, and we now make the definition of "true COI" more explicit and only refer to "true COI" when discussing simulations.

We agree that comparing the two methods against the same simulated data would be useful. We have added these as a new analysis to Appendix L.1.1 in the section titled: "Comparison to *THE REAL McCOIL*." These detail how the accuracy and bias of each method (both our methods and *THE REAL McCOIL)* perform when tested against the same simulated data.

5. *The claim that coiaf is more efficient but comparable to THE REAL McCOIL seems to be based on point estimates. When CIs with comparable coverage are also sought after, how much bootstrapping is required beyond the default 100, and how does that impact relative speed and memory in practice?*

Response: Thank you for raising this concern, which was also raised by reviewer #2. We agree that comparing point estimates is an unfair comparison. We have since revised this discussion to include the run time for our software when generating a confidence interval using bootstrapping. We also included a discussion of the results in Appendix I. We found that the speed of the point estimate generated by the discrete and continuous Variant Methods of coiaf remained constant even as the number of loci and samples increased. The speed of the bootstrapped method, the bootstrapped method when parallelization was used, and *THE REAL McCOIL* all increased in a seemingly linear fashion. However, the rate at which the run time of

our methods increased was significantly lower than the rate at which the run time of T*HE REAL McCOIL* increased. It is important to note that making direct comparisons is still difficult and depends on how many MCMC iterations are required for *THE REAL McCOIL* to converge and how many bootstrap replicates for *coiaf* are sufficient to correctly capture the uncertainty in COI. We have added a short discussion of these findings to the Discussion, with the benchmarking added as an additional figure in the Benchmarks Appendix I section.

To address the second point regarding how many replicates are needed, we explored how changing the number of replicates altered the standard error when estimating the COI on simulated data. We found that past the default value of 100, there was little change in the standard error. Arguably fewer than 100 replicates could be used, but for the sake of comparisons against *THE REAL McCOIL*, we used 100 replicates throughout. These findings have been added to the Results.

6. *It seems risky to me to provide an alternative method (Appendix D) that is seemingly untested: Is the alternative method validated? How does it compare with methods in the main text? What is the rationale for retaining q here and not in the objective functions in the main text?*

Response: Thank you for your suggestion. This alternative method outlined our original approach for estimating the complexity of infection, where we binned our data into groups before solving the optimization problem. We believe our current approach, which utilizes all data points, is simpler. We agree that providing this alternative method may confuse the reader, so we have removed this section from the appendix.

Minor Comments

1. *For clarity, could consider writing both equations (2) and (3) as expectations, i.e., E[Vi] = Pr(Vi = 1) since Bernoulli.*

Response: Thank you for your suggestion. We have added this to equation (2) to improve clarity.

2. *Line 30, co-transmission and/or superinfection, since they are not mutually exclusive.*

Response: Thank you for the comment. Lines 27-32 have now been updated to reflect this fact. They now read: “These polyclonal infections may arise from one or both of the following: (i) a single infectious mosquito feeds on a human host, transferring several genetically distinct parasite strains (often referred to as a co-transmission event) or (ii) two or more infectious mosquitoes with distinct malaria strains feed on an individual (known as a superinfection event)”

3. *Lines 87-95, I don’t see the value of defining reference allele frequencies. Moreover, there are several mistakes in the equations (see below) and the hat versus no hat notation for reference versus minor is misleading since it is usually used for parameter*

estimate versus parameter. Mistakes: the inline equation on line 95 compares a vector to a bold scaler and sums over loci when presumable summation is over samples; equation (1) also compares vectors to scalers and only lists the two extremes of the 2l cases: those where all reference PLAF are either minor or major.

Response: Thank you for your comment and for identifying mistakes in our equations. We feel that the referenced equations can be useful to users as, in most cases, they will have reference allele frequencies and will need to convert them to minor allele frequencies. This capability is already provided in our package, and we have updated the documentation to clarify this. However, we agree that for the main text, this conversion is not necessary. As a result, we have shortened the paragraphs defining the population-level and within-sample minor allele frequencies and moved the corrected equations to Appendix A.

4. *Line 107, presumably di is the within-sample coverage?*

Response: Thank you for your comment. You are indeed correct that di is the within-sample coverage. We have clarified this in the text on line 121, which states "... di is the within-sample coverage at locus i…".

5. *Unlike equations 19 and 20 in Appendix D, the norm parameter q doesn't feature in equations (4) and (6) albeit referred to in the text below. Is it mistakenly referred to or is it mistakenly omitted from equations (4) and (6)? And if omitted, is q = 2 fixed or a default value?*

Response*:* Thank you for highlighting this, which was also raised by reviewer #2. The value q was a remnant of an older formulation of our equations, and it has been removed from equations (4) and (6).

6. *Line 170, reserve k for COI.*

Response*:* Thank you for your comment. We have removed the reference to *k* groups.

7. *Fig 3C: define difference, given not absolute; why are most differences negative for the variant method and positive for frequency?*

Response*:* Thank you for highlighting this. We have added that this difference is calculated as *coiaf* - *THE REAL McCOIL* to the legend. We have also discussed in the legend why the differences are negative/positive for each method, which is largely due to the Frequency method being undefined for COI < 2. The high density observed above 0 for the Frequency Method occurs because the Frequency method is undefined for COI < 2. Consequently, for samples that *THE REAL McCOIL* estimates as having a COI equal to 2, the distribution of our estimates of COI using the Frequency method is skewed greater than 2, in contrast to the Variant method, which exhibits lower skewness.

8. *Fig 4C: add label to righthand axis.*

Response*:* Thank you for highlighting this. We have added a label to the righthand axis.

9. *Line 365-367: sampling bias and undersamping are not the only problems: to properly estimate allele frequencies, one would have to account for complex population structure that follows from co-transmission and superinfection.*

Response*:* Thank you for this comment. We agree that the spatial landscape of malaria transmission and heterogeneities in transmission intensities will lead to a complex spatial pattern of allele frequencies arising from different contributions of co-transmission and superinfection that will drive recombination and allele frequency fixation patterns. We have added this extra explanation to the discussion. Lines 423-426 now read: "Sampling bias, undersampling, or complex spatial patterns of allele frequencies resulting from the spatial landscape of malaria transmission and heterogeneities in transmission intensities in a region may result in an inaccurate PLMAF estimation, which may influence our estimated COI."

Reviewer #2

Major Comments

1. *The approach to estimate COI requires estimates of population level allele frequencies. The authors provide a nice proof that these may be estimated in an unbiased fashion from observed data. However, that is under the rather strong assumptions of no intrahost parasite relatedness and no error. The authors explore the impact of sequencing error and overdispersion of read counts which appear to demonstrate substantial sensitivity. However, it is difficult to interpret the meaning of the dispersion parameter as it would apply to a real world dataset. It would be enlightening for the authors to explore the impact of intrahost relatedness directly in their simulations. I bring this up specifically because THE REAL McCOIL demonstrates a great deal of robustness estimating allele frequencies, even under extreme degrees of intrahost relatedness.*

Response: Thank you for your comments that were shared with other reviewers. To help explain how the overdispersion in sequence read counts works in practice, we have added an additional figure to the Supplement (G.1 Simulator Parameters, Figure 1) to graphically explain how overdispersion in read counts will impact sequencing. This also includes a figure to explain how overdispersion in parasite density impacts sequencing.

In relation to the impact of relatedness, we have included intrahost relatedness between strains to our simulator and have explored this in the Sensitivity Analysis (Appendix L.1, Sensitivity analysis, Figure 9) and also in our new set of simulations where we do direct head to head comparisons between *THE REAL McCOIL* and our methods against simulated data (Appendix L.1.1., Figure 4). These both show how increasing intrahost relatedness downward biases our estimates of COI. This impact is comparable to *THE REAL McCOIL*, with the same decrease in estimated COI with increasing relatedness. These findings have been discussed in the Results and Discussion section.

Lastly, we also include new head-to-head analyses that compare *THE REAL McCOIL*'s estimation of allele frequencies against our estimates (Appendix L.1.2). These show equally strong performance at predicting allele frequencies. This is also confirmed in the appendix where we derive that using continuous measures of within-sample allele frequencies, i.e. how many read counts are from one allele over the total read count as opposed to simply whether a locus is heterozygous, gives an unbiased estimate of the population allele frequency by simply averaging over the population's within sample allele frequencies (Appendix C.1, An unbiased estimator of the PLMAF).

2. *The claimed benefit of computational efficiency is somewhat overstated. While a single instance of running the algorithm may be fast, the authors themselves state that bootstrapping is required to evaluate the uncertainty of estimates. Although this can certainly be parallelized, it still does not warrant the claims of computational superiority as stated. Assuming perfect linear speedup of multiprocessing, bootstrapping 1000*

samples would require a quadcore processor to achieve parity with THE REAL McCOIL alone.

Response: Thank you for raising this concern, which was also raised by reviewer #1. We have included our response to their question below, which details how we have added additional benchmarking comparisons by comparing bootstrapped estimates using our methods. In our appendix, we also explore how the standard error in estimates of COI asymptotes with increasing bootstrap replicates.

Response to reviewer #1:
We agree that comparing point estimates is an unfair comparison. We have since revised this discussion to include the run time for our software when generating a confidence interval using bootstrapping. We also included a discussion of the results in the Appendix I. We found that the speed of the point estimate generated by the discrete and continuous Variant Methods of coiaf remained constant even as the number of loci and samples increased. The speed of the bootstrapped method, the bootstrapped method when parallelization was used, and *THE REAL McCOIL* all increased in a seemingly linear fashion. However, the rate at which the run time of our methods increased was significantly lower than the rate at which the run time of T*HE REAL McCOIL* increased. It is important to note that making direct comparisons is still difficult and depends on how many MCMC iterations are required for *THE REAL McCOIL* to converge and how many bootstrap replicates for coiaf are sufficient to correctly capture the uncertainty in COI. We have added a short discussion of these findings to the Discussion, with the benchmarking added as an additional Figure in the Benchmarks Appendix I section.

3. *Coiaf is directly compared to THE REAL McCOIL. However, the manuscript lacks any direct comparisons under the specified simulations, making it challenging to reconcile the differences observed within the real data application. Differences in accuracy should be compared directly under a gold standard setting to explore the limitations of both models.*

Response: Thank you for raising your concern about comparing *coiaf* to *THE REAL McCOIL*—the same point was raised by both the other reviewers. We agree that comparing the two methods against the same simulated data would be useful. We have added these as a new analysis in the Sensitivity Analysis results section titled: "Comparison to *THE REAL McCOIL*." These detail how the accuracy and bias of each method (both our methods and *THE REAL McCOIL)* perform when tested against the same simulated data. We included five separate analyses, varying the coverage, number of loci, level of overdispersion in parasite density, relatedness between parasite strains, and the error introduced into our simulation. Across each analysis, we found few differences between our software and *THE REAL McCOIL*. As the number of loci and the coverage increased, the performance of both software packages improved. Conversely, as greater levels of overdispersion, relatedness, and sequence error were introduced into the simulations, both methods underpredicted the true COI. We have added a description of these findings to the Results and the Discussion.

Minor Comments

1. *The expression for PLAF in line 95, I believe should be indexed by n, not l (l is already used to index loci)*

Response: Thank you for highlighting this error. The expression should indeed be indexed by n instead of l, which we have corrected. We have also decided to move this section to Appendix A as it induced added complexity, as pointed out by reviewer #1. In the main text, we now begin the problem formulation by defining the PLMAF and the WSMAF.

2. *The authors make reference to a value q following equations 4 and 6, but is undefined. Perhaps, it's supposed to be k?*

Response: Thank you for your comment, which was also raised by reviewer #1. The value q was a remnant of an older formulation of our equations, and its reference has been removed from the text.

Reviewer #3

Major Comments

1. *Currently, the comparison between TheRealMcCoil and coiaf is limited to sequence data from a set of globally distributed samples. The two methods perform similarly but not identically, and I am left wondering how the accuracy of the two approaches compares. I suggest that the authors include a head-to-head comparison of coiaf and TheRealMcCoil on the simulated data as well, showing both COI and population level allele frequency estimates for the two methods.*

Response: Thank you for raising your concern about comparing coiaf to *THE REAL McCOIL*—the same point was raised by both the other reviewers. We agree that comparing the two methods against the same simulated data would be useful. We have added these as a new analysis in the Sensitivity Analysis results section titled: "Comparison to *THE REAL McCOIL*." These detail how the accuracy and bias of each method (both our methods and *THE REAL McCOIL*) perform when tested against the same simulated data, both for estimating COI and allele frequency. We included five separate analyses, varying the coverage, number of loci, level of overdispersion in parasite density, relatedness between parasite strains, and the error introduced into our simulation. Across each analysis, we found few differences between our software and *THE REAL McCOIL*. As the number of loci and the coverage increased, the performance of both software packages improved. Conversely, as greater levels of overdispersion, relatedness, and sequence error were introduced into the simulations, both methods underpredicted the true COI. We have added a description of these findings to the Results and the Discussion.

2. *In regards to assessing accuracy, I also suggest that the authors expand the simulated data results presented in the main text. I appreciated the wider range of parameters explored in the Supplement, and readers would benefit from seeing these data in the main results. In particular, I think it is worth highlighting the effects of strain proportions, read depth, and overdispersion of read counts.*

Response: Thank you for your interest in the sensitivity analyses. We agree we need to discuss these sensitivity analyses in the main text. The read depth is described in the second paragraph of the Sensitivity Analysis Results section. We have also added an additional paragraph to the Sensitivity Analysis section of the main text, where we explore the impact of intrahost relatedness (a new parameter explored in response to all reviewers raising this need) and strain proportions and overdispersion of read counts.

3. *Fig 1 in the appendix shows that coiaf produces an unbiased estimator of population level allele frequencies, however, accuracy is low, especially in the region of typical sample sizes (<=100). I would like to see these estimates compared to those produced using current common approaches: (1) estimating allele frequencies from putatively monoclonal samples only and (2) using TheRealMcCoil. Whether highly accurate maf*

estimates are necessary for COI estimation is another matter. The authors could demonstrate that their method provides sufficient accuracy for estimating COI without the added computational expense of accurately estimating population mafs. In this case, however, I would suggest they clarify this in the text.

Response: Thank you for the comment. For clarification, *coiaf* estimates the PLAF as the mean of all within-sample allele frequencies across each locus. This is now discussed in the appendix where we derive that using continuous measures of within-sample allele frequencies, i.e., how many read counts are from one allele over the total read count as opposed to simply whether a locus is heterozygous, gives an unbiased estimate of the population allele frequency by simply averaging over the population's within sample allele frequencies (Appendix C.1, An unbiased estimator of the PLMAF). We have highlighted and clarified this mathematical finding in our main text and in the conclusion of the Discussion as we feel this itself is a key finding and makes a case for using continuous estimates of allele read depth to estimate population-level allele frequency.

We have also added an additional figure to the supplement related to the new analyses in which we compare *THE REAL McCOIL* against *coiaf* on simulated data, where we show the estimated PLAF by both approaches you have detailed. This shows that each method provides accurate estimates of the true PLAF. We have referred to these results now in the Results and Discussion, where we discuss that if within-sample allele frequency estimates are available (as opposed to just homo/het calls) that PLAF estimation is not required concomitantly with COI. However, it may still be helpful to do this if the number of samples is small (<25). Alternatively, you can use a priori known PLAF., e.g., estimated from global databases such as Pf6k.

4. *In the absence of evidence that coiaf is more accurate than TheRealMcCoil, coiaf's increased computational efficiency strikes me as the most compelling benefit over the other. I would suggest highlighting this in the main Results (with a figure even). The authors might also consider giving increased emphasis to coiaf's ability to handle whole-genome data, although given the diminishing returns observed after crossing the 1000 loci threshold, this may not be a major benefit.*

Response: Thank you for your suggestion. We have added a more in-depth discussion of these speed comparisons to the main results. We have additionally added a new speed comparison between the two methods to include the run time for our software when generating a confidence interval using bootstrapping. We have added a short discussion of these findings to the Discussion, with the benchmarking added as an additional figure in Appendix I, Benchmarks. In addition, we have also discussed the advantages of having a method that can provide a quick point estimate, i.e., without bootstraps, as well as exploring the uncertainty as we have with the Bootstrap approach (Appendix F, Convergence of bootstrap replicates).

Minor Comments

1. *The authors state that continuous COI estimates can provide insight into the relatedness of parasites in complex infections. In theory, I agree with this; in practice, I do not know how easy it is to interpret such numbers vis-a-vis other factors like low read coverage and genotyping error. Simulating and analyzing data to this effect would be a useful addition to this manuscript as I agree that this is an area of interest for the field.*

Response: Thank you for the comment—this is an area we are particularly interested in. We plan to explore this in an additional study because we felt it important to generate sequence data that is biologically realistic and represents the peculiarities of the malaria life cycle. In response, we hope to use one of the more realistic malaria genetic simulators (Watson et al. MBE 2021, SIMPLEGEN (https://mrc-ide.github.io/SIMPLEGEN/index.html) or the IDM model) to capture this. In addition, we felt that to look at this, as you point out, we would need a more sophisticated method for simulating the sequencing process from known samples (rather than a single error parameter for incorrect read calls) and pairing this with more believable read coverage. As a result, we feel this is a new study entirely—although we largely agree that gaining interpretable information may be unlikely in reality and under common sequencing/sampling constraints.

2. *In Fig 1, the points are so dense that it is hard to derive any information from these plots. If the goal is to visualize the analytical steps, a cartoon schematic might be easier to interpret. (This is just a suggestion)*

Response: Thank you for this comment. We have added color to the plots to clarify which data points are used in which method, i.e., which loci are variants. We hope this has made the figure more interpretable.

3. *The DePloidIBD paper (https://doi.org/10.7554/eLife.40845) does include information on the global distribution of Ke ('effective number of strains'). While this isn't equal to COI in a strict sense, it is highly related. These results could be compared to the global COI estimates made here.*

Response: This is a great idea, and we completely agree. We have added this comparison to Appendix L.3, Figure 4 along with the FwS comparison, which again shows a strong correlation. Thank you for the suggestion.

4. *It appears the simulations assume all loci are unlinked. This is a reasonable and common assumption, but I suggest making this explicit in the text since WGS and some targeted sequencing approaches generate linked variants. (I did see that LD was addressed in the appendix in regards to running TheRealMcCoil.)*

Response: Thank you for raising this point which reviewer #1 raised. We have clarified these assumptions and explained how they impact our methods if violated at the end of the Variant

Method section in the Methods. Namely, if violated, the variance in estimates generated by both methods will be greater, but the bias will remain unchanged. Additionally, an in-depth explanation of this has been added to Appendix C.2, Impact of correlation among loci. Please note that *COIL* arrives at the same result in their original paper, and arguably the supplementary analysis in *THE REAL McCOIL* paper that looks at this only shows a small impact on the trends shown in sensitivity analyses).

5. *Many genotyping approaches now rely on a pre-amplification step prior to genotyping/sequencing. The (potential) effects of this could be mentioned in the Discussion.*

Response: Thank you for this comment. It's an important factor that likely leads to the amplification of higher parasitemia samples and may result in lower COI estimates inferred from the resulting sequence data. We have added this to the limitations section of the discussion. Lines 420-421 read: "The amplification of higher parasitemia samples may obscure lower prevalence parasite strains and result in lower inferred COI estimates."

6. *Do you suggest that users take the COI probabilities into account? Does some filtration at this level increase accuracy?*

Response: The probabilities presented along with the discrete method give the probability density function for each COI for a sample, which is provided mostly to show if a COI estimate is confident on one particular COI or another. We developed this approach first before developing the bootstrapped approach, which we feel is a better method to approximate the uncertainty but one that takes more computational time than the COI probabilities. Both are available, though, to give users with different needs options. An efficient approach may be to run the discrete approach and assess if the probability density is shared significantly across multiple COI values, in which case the bootstrapped continuous could be used to better estimate the uncertainty in COI. We hope this gives more information about the COI probabilities.

7. *Issues with GitHub links, pages, and suggestions:*
    a. *- On the GitHub page, the links to the vignettes are broken*
    b. *- A simple manual would be a helpful addition. For instance, the text mentions incorporating genotyping error into the model, but it was unclear from the examples how to do this short of editing the main code. I also never found the code used for bootstrapping CIs.*
    c. *- Running the 'method 2, continuous' example in 'example_real_data.Rmd', all COI values were over-inflated (including many with COI=25)*
    d. *I found a few small errors in 'example_real_data.Rmd' (NB: I was using the latest release, not the dev version)*
    e. *line 57, 85, 100, 128: a character string [0,1] is requested for coi_method*
    f. *line 56, 84, 99, 127: expected tibble values were wsaf and plaf*

Response: Thank you very much for taking the time to go through the package and examples so thoroughly and for improving the interpretability of the package documentation. We have addressed all the above comments on the package under the new release (v0.1.1), which we hope is clearer and easier to use. Thank you again—we really appreciate it.
