# Supplementary material for: coiaf: Directly estimating complexity of infection with allele frequencies
Source: PLoS Comput Biol. 2023 Jun 9;19(6):e1010247. doi: 10.1371/journal.pcbi.1010247 (PMC10310041; doi:10.1371/journal.pcbi.1010247)
Supplement: Attachment — Submitted filename: Response to Reviewers.pdf [file pcbi.1010247.s005.pdf]

Dear Reviewers,

We would like to express our gratitude for your time and effort in reviewing our manuscript and providing insightful comments and suggestions. Your insightful feedback has greatly improved the quality and clarity of our work, and we appreciate your dedication to ensuring the highest standards of scientific rigor.

Based on your comments, we have further clarified and improved several aspects of our manuscript. We hope you will find the revised manuscript a satisfactory response to your comments.

Reviewer #1

The authors have gone to great efforts to revise their manuscript, which is much improved. I still have some outstanding concerns, but they are hopefully quick to fix.

Major Comments

1. *Independence assumption:*

   I appreciate the authors' efforts to clarify the independence assumption. However, I still find it misleading to state that markers in LD are unsuitable for THE REAL McCOIL without equal mention of coiaf (Appendix K), and I believe my original comment on independence/variance has been interpreted incorrectly (see below). Also, p of equation 14 (Appendix C.2) is not defined (personally, I don't find the addition of equation 14 very helpful).

   Previously I wrote, "Under a model that captures the finite nature of the genome, the reduction in variance with increasing loci count would asymptote." To which, the authors responded, "This reduction in variance will eventually asymptote as the number of loci included tends towards the total number of variant sites in the genome." This is incorrect. The coiaf model does not account for the finite nature of the genome. When loci are treated independently (e.g., coiaf), the model is liable to have an increasingly false sense of confidence with added loci, because it does not account for the fact that as more and more loci are added, they get more and more dependent and thus less and less informative. As such, confidence intervals are liable to be over-optimistically narrow (i.e., have coverage less than 100(1 - alpha) %) and the situation will get worse and worse as the density of loci on the finite-length genome increases. As such, I would expect the variance under coiaf to get smaller with each added locus at a constant rate and the n just stop when there are no more loci to add. Whereas, preferably (under a dependence model), the reduction in variance would decelerate as added loci are less and less informative. I suggest the authors revise lines 112-113, 235-236, Appendix C.2, etc., accordingly, e.g., at lines 112-113, I would have said "if the independent loci assumption is not met, the confidence intervals around COI estimates will likely have less coverage than stated, but the bias in estimated COI should not change, providing the model is otherwise well specified."*

Response: Thank you for your comment. We apologize for misunderstanding your original comment on the effect of independence on variance. You make two different but valid points here:

1. Including non-independent loci will impact the estimator's variance.
2. Increasing the number of loci sampled will eventually require including loci that are not independent.

We fully agree with both of these points. To show the first point, we conducted an additional analysis where we compared the distribution of point estimates of COI between data sampled from completely independent loci and data sampled from a set of dependent loci. In this analysis, we observe that the bootstrapped confidence interval is wider when using linked loci than when using non-independent loci. We have included this explanation in the revised lines 112-114 of the main text, as you have suggested, stating:

"If this assumption is not met, the confidence intervals around COI estimates will be impacted with bootstrapped confidence intervals increasing, but the bias in estimated COI should not change, providing the model is otherwise well specified (see Appendix C.2 in S1 Appendices)."

With regard to the impact of increasing the number of loci, assuming these loci are independent, this will decrease the variance in our estimator. However, as this tends to the full extent of the genome, new loci will be increasingly sampled from sites that are not independent of already selected loci, and this will thus impact the variance. We have explained this now in the revised lines 233-235:

"This reduction in variance will continue to decrease as the number of loci included increases. However, this is dependent on whether loci are independent of one another (see Appendix C.2 Fig 1)."

In Appendix C.2, we include an extra paragraph describing our new analysis where we compare the impact of independence on the variance. We write:

"The variance in our estimator, on the other hand, will increase as we do not account for the fact that non-independent loci are less informative than dependent loci. In Fig 1 (SI Section C2), we compare the distribution of point estimates for two data sets across several COIs. Data were sampled from 1,000 independent loci to generate the first data set. To generate the second data set, we first sampled from 500 independent loci and subsequently duplicated these data points—yielding a set of 1,000 dependent loci. We observe that the variance in estimated COI is smaller when sampling from the set of independent loci."

Lastly, we apologize for the confusion caused by equation 14. p was meant to be p_i , i.e., the population-level minor allele frequency (PLMAF) at locus *i*. We have removed the equation as we agree it was unhelpful.

3. *Bootstrap replicates and comparison with THE REAL McCOIL:*

   For a fair comparison of the run time of bootstrap-coaif and THE REAL McCOIL, as summarised in lines 341 onwards, I believe it is necessary to first show that the number of chosen bootstrap replicates (100) provides confidence intervals whose coverage are comparable to that of the credible intervals of THE REAL McCOIL, at least for some of the test cases used to generate Fig 3 of Appendix I. As an aside, based on Appendix F, I

would have said at least 200 bootstraps are needed for the stable regime, at least in so far as the COI 5 and 1,000 loci test case is concerned.

Response: Thank you for your comment. To further support our choice of 100 replicates as the default threshold, we ran additional simulations examining the standard deviation as the number of replicates changes for a larger set of COIs and plotted the results. The plot, which serves to augment the original figure in Appendix F, illustrates that there is decreasing variance with increasing replicates, with the variance with 100 replicates largely comparable to the variance with 1,000 replicates. Additionally, we agree that it is important to illustrate that the confidence interval between the two packages is comparable to our chosen default value. As such, we have added a figure to Appendix I, Benchmarks, which shows that the distribution of point estimates for THE REAL McCOIL and bootstrapped coiaf are similar.

4. *PLMAF estimation:*

   The authors show that the mean WSMAF is an unbiased estimate of the PLMAF. This is only true if the s_{ij} are independent of the C_{ij} (equation 7 of Appendix B.3), as is the case in the simulations, but not in the case of loci under drug-selection post treatment, for example. This implicit assumption needs explicitly stating in the appendix (equation 7 of Appendix B.3 and Appendix C.1, instead of "regardless of any assumptions about the distribution of strain proportions") and in the main text where estimation of the PLMAF is mentioned.

Response: Thank you for your comment. We agree with you that this implicit assumption holds and that it should be stated that we presume the vast majority of markers are neutral where selection is not dramatically rapidly altering allele frequencies. We have revised the text following this derivation in B.3 to include the lines:

"However, this will be a biased estimator of p_i if $s_{i,j}$ are not independent of $C_{i,j}$. For instance, if $\boldsymbol{C}_i$ is associated with drug resistance and sample collection was biased to reveal this non-independence, i.e., samples were collected from individuals after drug treatment, increasing resistant parasite strains, our estimator would differ between the treated and untreated malaria population."

And in Appendix C.1:

"We note that this assumes no sequencing error and that sample collection is independent of strain proportions."

Additionally, in the main text, we have added lines 355-357 stating:

"This finding requires certain assumptions which may not be met, for example, for loci associated with drug resistance and when sampling selectively from individuals following drug treatment."

Overall, a few loci under selection are unlikely to greatly affect the overall estimate of COI, presuming the vast majority of loci are not being altered by treatment or other conditions, but we agree that violating this assumption will impact the bias in our estimator of p_i if the sampling of individuals is biased.

Minor Comments

1. *Equation 4, consider v_i instead of indicator function*

Response: Thank you for the recommendation. We have replaced the indicator function with v_i.

2. *Line 195 and Table 1 of appendix H, read depth is 100 and 200, resp. (inconsistent).*

Response: Thank you for pointing out this inconsistency in our read depth. In our sensitivity analysis, we simulated data with a read depth of 200. Table 1 of Appendix H correctly reflects this value. As such, we have changed Figure 2 and the main text to illustrate data with a read depth of 200.

3. *Lines 226, the relationship is not linear, but it seems it is monotonic.*

Response: Thank you. We have changed the text stating that the relationship is monotonic and non-linear.

4. *Appendix B.3, $\boldsymbol{s}_i$ appears to be treated as a non-random fixed variable, but surely it is not observable (only the aggregate reads for the minor / major are observable, no?).*

Response: You are indeed correct that $\boldsymbol{s}_i$ is non-observable. We have removed the word observable from the text.

5. *Appendix D, second inequality of the last sentence, 1 > (1-WSMAF) >= 1 epsilon?*

Response: Thank you. We have fixed the inequality to read “1 - WSMAF”.

6. *Appendix G, tau = S x G has an unnecessary subscript; tau is in interval [0,0.5)? S is in the simplex? G is in {0,1}?*

Response: The subscript for G_i is indeed unnecessary. We have removed the subscript from the equation.

7. *Appendix G, how exactly do the parameters of the beta-Binomial relate to the over-dispersion values in Fig 1B in Figures 8&9 of Appendix L? Values seem to be on very different scales, such that Fig 1B does not aid interpretation of Figs 8&9.*

Response: We apologize for this error and thank you for pointing this out. There were two errors on our side here as we were using the incorrect way of parameterising the beta binomial when producing Fig 1B and we were not using values of overdispersion that were on a helpful scale for interpretation (we would have caught this if we had been!). We have corrected this to now use the correct form for overdispersion, as used by our simulator. We, furthermore, now show the distributions for values of overdispersion as used in Figure 8 of Appendix L.1 (i.e., 0, 0.05, 0.1, 0.15, 0.2, 0.25). Please see the resulting figure below, which is also in Appendix G:

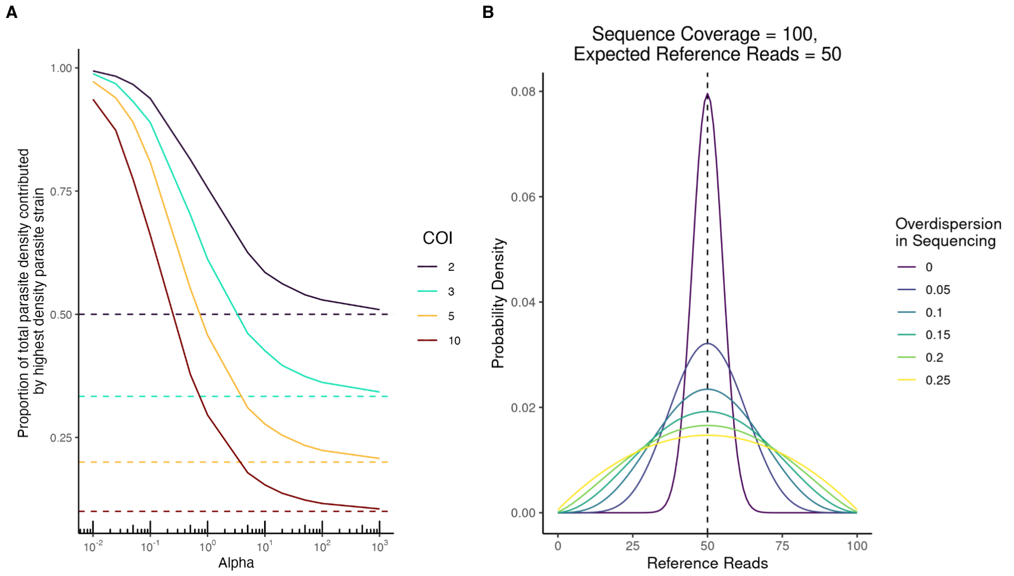

8. *Appendix L.1.1, Figure 5, are plot colours correct for panels B and C? Switch in bias performance seems bizarre.*

Response: Thank you for raising this concern. First, we regret that we mislabeled the title and subplot titles of the figure, which has now been fixed. Appendix L.1.1, Figure 5 shows the change in performance as epsilon, the probability of a single read being miscalled as the other allele in our simulations, changes. With regards to the change in bias between panels B and C, this stems from the fact that we let both THE REAL McCOIL and coiaf estimate the amount of error believed to be present in the simulations, defined by the parameter sequence error. As the error of each three panels is estimated separately, there can be a difference in the bias, depending on whether the estimated sequence error was larger or smaller than the true error introduced into our simulations. We have updated the figure legend to clarify how error is treated.

9. *Figure 1 main text and Figure 2 appendix L.2 are missing colour legends.*

Response: Thank you for your comment. We have added color legends to Figure 1 of the main text and Figure 2 of Appendix L.2.

10. *Appendix L.3 Fig 6, unclear to me what is meant by assumed sequencing error. Did you let both The Real McCOIL and coiaf infer the error? That would seem the fairest way to compare the two approaches.*

Response: Thank you for your comment. We apologize for the confusion. Appendix L.3 Figure 6 aims to show how the results of our estimation method can drastically change depending on the level of sequence error that is assumed in the population. The graph is not meant to provide a head-to-head comparison of the two software packages. We have updated the figure caption to clarify what is being plotted.

Reviewer #2

The authors have sufficiently addressed my major concerns through direct comparisons to THE REAL McCOIL on simulated data. I only have minor comments regarding some of the supplementary figures and minor copy editing.

Minor Comments

1. *Line 118 appears to have the beginning cut off.*

Response: Thank you for catching this. The beginning of the line now reads: “Given data, *D…”*.

2. *In figures comparing performance with THE REAL McCOIL using simulated data, how many bootstrapped samples are being used to estimate 95% CI for coiaf methods? If it's not default setting of 100, then this should be mentioned and the plots comparing speeds should be done with the same number of bootstrapped samples*

Response: Thank you for your comment. In general, for figures comparing the performance of THE REAL McCOIL and coiaf using simulated data, we are not using the bootstrapped methods. When comparing the run time between our bootstrapped methods and THE REAL McCOIL, we use the default number of replicates of 100; we have ensured this is stated in the legend text. We have, moreover, included some additional information in Appendix F to support the value of 100 as the default number of replicates.

3. *It's unclear to me the difference between epsilon and sequencing error in simulations. I see that epsilon is varied when evaluating the performance of the different estimation approaches and the figure text (L.1 fig 10) explains that sequencing error is fixed at 1%. However, in the comparisons to THE REAL McCOIL (L.1.1 fig 5), the text states that the sequencing error is varied, is this distinct from varying epsilon? I ask because the values chosen varying sequencing error when comparing to THE REAL McCOIL match the values of epsilon varied earlier (0.05, 0.01, 0.015), however the performance is wildly different for coiaf between the two figures. This same concern applies to estimating PLAF as well, but that is not evaluated earlier.*

Response: Thank you for raising this concern. We acknowledge that the definitions of epsilon and sequence error may be confusing in their current form. To clarify, epsilon is the probability of a single read being miscalled as the other allele in our simulations. Sequence error, on the other hand, is the level of sequence error that is assumed in our estimation methods (see Appendix D for how we account for this error in our methods). In other words, epsilon is used when simulating data, whereas sequence error is used when estimating the COI and serves to estimate the true simulated error, epsilon. To clarify this distinction, we have revised the definitions of these parameters in Table 1 from Appendix H.

With regards to Appendix L.1, Figure 10 and Appendix L.1.1, Figure 5, both figures show the performance of the estimation methods as the value of epsilon changes. We apologize for the confusion. We have changed the title and subplot titles of Appendix L.1, Figure 10, Appendix L.1.1, Figure 5 (and the related Appendix L.1.2, Figure 5) to clarify this. We have added text to the figure legends to clarify the error being varied. The reason for the difference in performance between Appendix L.1, Figure 10 and Appendix L.1.1, Figure 5 stems from the amount of error assumed in the estimation methods, known as sequence error. In Appendix L.1, Figure 10, the amount of error assumed is fixed at 1%. However, in Appendix L.1.1, Figure 5, we let coiaf estimate the amount of error. We have updated the figure legends to make this clearer.

Reviewer #3

In this revision, Paschalidis and colleagues have given thoughtful consideration to reviewers' comments and substantially strengthened this version. In my opinion, this current manuscript clearly presents sufficient detail for readers to understand and evaluate this new COI estimation method in relation to currently used approaches.

Response: Thank you very much for your comment. We have greatly appreciated your constructive feedback in improving our manuscript.
