# Supplementary material for: coiaf: Directly estimating complexity of infection with allele frequencies
Source: PLoS Comput Biol. 2023 Jun 9;19(6):e1010247. doi: 10.1371/journal.pcbi.1010247 (PMC10310041; doi:10.1371/journal.pcbi.1010247)
Supplement: Attachment — Submitted filename: coiaf.docx [file pcbi.1010247.s002.docx]

Malaria infections can contain genetically distinct parasite strains. The number of genetically distinct strains within an infection (the complexity of infection, COI) is an important target of inference in malaria genomic epidemiology for various reasons. Several methods exist for estimating COIs using parasite genetic data. This manuscript details a new approach (coiaf) that claims to be more efficient than the current gold standard (THE REAL McCOIL: a Bayesian model, implemented using MCMC). coiaf supports data on biallelic SNPs. It returns the COI that minimises the sum (over SNPs) of the squared weighted residuals between observed and expected values of SNP-wise data summaries, where residuals are weighted such that SNPs with higher coverage contribute more to optimisation. Two data summaries are proposed in the main text: for the *i*-th SNP, read data can either be summarised as homo or heteroallelic (*v_i_* = 0 or 1, respectively) or as a minor allele frequency (*w_i_*). Expectations are derived under a model that assumes (1) loci are independent and that (2) strains within complex infections (those with COI > 1) are unrelated. Data are simulated under the same assumptions. Sequencing errors are accounted for when computing data summaries, i.e., they are pre-processed. coiaf is validated using simulated data. Its utility is demonstrated using data on *P. falciparum*, which are also analysed using THE REAL McCOIL for comparison.

This manuscript is clearly and concisely written. The claims made are largely supported by the results. However, I do have a few concerns:

1. Formalisation of the objective functions: Usually in weighted least squares, the weighted sum of squared residuals is minimised (e.g. see page 389 of Gelman and Hill, *Data analysis using regression and multilevel/hierarchical models*), not the sum of weighted residuals squared (coiaf). As such, the COI estimate of coiaf is not the MLE of a weighted regression, but something else which I don’t quite understand. This irregular approach might explain the non-linear relationship between coverage and performance (line 212) and the poor coverage of confidence intervals (Appendix E and plots A.3. and B.3 of Figs 4 and 5 of Appendix K), which I’m also struggling to understand. It would make more sense to do regular weighted least squares. At the very least, the authors should 1) verify numerically that this alternative estimator is consistent, i.e., for a fixed large COI, 20 say, show that as the number of data points increases (x axis) the estimates (y axis) converge to the true COI; and 2) include a caveat highlighting that the approach is irregular.
2. Model assumptions: The assumptions of the underlying model (see summary above) could be better articulated in the main text (e.g in Materials and methods and again on line 355). Indeed, their implicit nature appears to be misleading:
   a. Appendix J details how SNPs in LD are unsuitable for THE REAL McCOIL; however, the same is true of coiaf, given assumption 1.
   b. Under a model that captures the finite nature of the genome, the reduction in variance with increasing loci count (line 221) would asymptote.
3. Misspecification: how does coiaf perform when the two model assumptions (independent loci and unrelated strains) are violated? For a regular MLE, the first is likely to impact CI coverage but not the point estimates, since correlation among SNPs simply reduces the information content in the data.

1. Benchmarking: I am confused about which data were used to benchmark coiaf and THE REAL McCOIL (sorry if I’ve missed something). The main text mainly implies COI estimates from the pf samples with unknown true COIs were compared, but reference to the “true COI” (line 240) suggests otherwise. Were pf data engineered in silico to generate complex pf infections with known COI? Fig 5 of Appendix K.3 implies some bench marking was done using simulated data. In any case, I expected to see plots that compare error and CI coverage across the two methods.
4. The claim that coiaf is more efficient but comparable to THE REAL McCOIL seems to be based on point estimates. When CIs with comparable coverage are also sought after, how much bootstrapping is required beyond the default 100 and how does that impact relative speed and memory in practice?
5. It seems risky to me to provide an alternative method (Appendix D) that is seemingly untested: Is the alternative method validated? How does it compare with methods in the main text? What is the rational for retaining *q* here and not in the objective functions in the main text?

Minor comments

2. For clarity, could consider writing both equations (2) and (3) as expectations, i.e., E[Vi] = Pr(Vi = 1) since Bernoulli.
3. Line 30, co-transmission and/or superinfection, since they are not mutually exclusive.
4. Lines 87-95, I don’t see the value of defining reference allele frequencies. Moreover, there are several mistakes in the equations (see below) and the hat versus no hat notation for reference versus minor is misleading since it is usually used for parameter estimate versus parameter. Mistakes: the inline equation on line 95 compares a vector to a bold scaler and sums over loci when presumable summation is over samples; equation (1) also compares vectors to scalers and only lists the two extremes of the 2*^l^* cases: those where all reference PLAF are either minor or major.
5. Line 107, presumably *d_i_* is the within-sample coverage?
6. Unlike equations 19 and 20 in Appendix D, the norm parameter *q* doesn’t feature in equations (4) and (6) albeit referred to in the text below. Is it mistakenly referred to or is it mistakenly omitted from equations (4) and (6)? And if omitted, is *q* = 2 fixed or a default value?
7. Line 170, reserve *k* for COI.
8. Fig 3C: define difference, given not absolute; why are most differences negative for the variant method and positive for frequency?
9. Fig 4C: add label to righthand axis.
10. Line 365-367: sampling bias and undersamping are not the only problems: to properly estimate allele frequencies, one would have to account for complex population structure that follows from co-transmission and superinfection.
