# Supplementary material for: coiaf: Directly estimating complexity of infection with allele frequencies
Source: PLoS Comput Biol. 2023 Jun 9;19(6):e1010247. doi: 10.1371/journal.pcbi.1010247 (PMC10310041; doi:10.1371/journal.pcbi.1010247)
Supplement: Attachment — Submitted filename: revised_review.docx [file pcbi.1010247.s004.docx]

The authors have gone to great efforts to revise their manuscript, which is much improved. I still have some outstanding concerns, but they are hopefully quick to fix.

**Major concerns**

*1) Independence assumption*

I appreciate the authors’ efforts to clarify the independence assumption. However, I still find it misleading to state that markers in LD are unsuitable for THE REAL McCOIL without equal mention of coiaf (Appendix K), and I believe my original comment on independence/variance has been interpreted incorrectly (see below). Also, p of equation 14 (Appendix C.2) is not defined (personally, I don’t find the addition of equation 14 very helpful).

Previously I wrote, “Under a model that captures the finite nature of the genome, the reduction in variance with increasing loci count would asymptote.” To which, the authors responded, “This reduction in variance will eventually asymptote as the number of loci included tends towards the total number of variant sites in the genome.” This is incorrect. The coiaf model does not account for the finite nature of the genome. When loci are treated independently (e.g., coiaf), the model is liable to have an increasingly false sense of confidence with added loci, because it does not account for the fact that as more and more loci are added, they get more and more dependent and thus less and less informative. As such, confidence intervals are liable to be over-optimistically narrow (i.e., have coverage less than 100*(1 - alpha) %) and the situation will get worse and worse as the density of loci on the finite-length genome increases. As such, I would expect the variance under coiaf to get smaller with each added locus at a constant rate and the

n just stop when there are no more loci to add. Whereas, preferably (under a dependence model), the reduction in variance would decelerate as added loci are less and less informative. I suggest the authors revise lines 112-113, 235-236, Appendix C.2, etc., accordingly, e.g., at lines 112-113, I would have said “if the independent loci assumption is not met, the confidence intervals around COI estimates will likely have less coverage than stated, but the bias in estimated COI should not change, providing the model is otherwise well specified.”

*2) Bootstrap replicates and comparison with THE REAL McCOIL*

For a fair comparison of the run time of bootstrap-coaif and THE REAL McCOIL, as summarised in lines 341 onwards, I believe it is necessary to first show that the number of chosen bootstrap replicates (100) provides confidence intervals whose coverage are comparable to that of the credible intervals of THE REAL McCOIL, at least for some of the test cases used to generate Fig 3 of Appendix I. As an aside, based on Appendix F, I would have said at least 200 bootstraps are needed for the stable regime, at least in so far as the COI 5 and 1,000 loci test case is concerned.

*3) PLMAF estimation*

The authors show that the mean WSMAF is an unbiased estimate of the PLMAF. This is only true if the *s_ij_* are independent of the *C_ij_* (equation 7 of Appendix B.3), as is the case in the simulations, but not in the case of loci under drug-selection post treatment, for example. This implicit assumption needs explicitly stating in the appendix (equation 7 of Appendix B.3 and Appendix C.1, instead of “regardless of any assumptions about the distribution of strain proportions”) and in the main text where estimation of the PLMAF is mentioned.

**Minor points**

1. Equation 4, consider *v_i_* instead of indicator function
2. Line 195 and Table 1 of appendix H, read depth is 100 and 200, resp. (inconsistent).
3. Lines 226, the relationship is not linear, but it seems it is monotonic.
4. Appendix B.3, ***s****_i_* appears to be treated as a non-random fixed variable, but surely it is not observable (only the aggregate reads for the minor / major are observable, no?).
5. Appendix D, second inequality of the last sentence, 1 > (1-WSMAF) >= 1 epsilon?
6. Appendix G, tau = S x G has an unnecessary subscript; tau is in interval [0,0.5)? S is in the simplex? G is in {0,1}?
7. Appendix G, how exactly do the parameters of the beta-Binomial relate to the over-dispersion values in Fig 1B in Figures 8&9 of Appendix L? Values seem to be on very different scales, such that Fig 1B does not aid interpretation of Figs 8&9.
8. Appendix L.1.1, Figure 5, are plot colours correct for panels B and C? Switch in bias performance seems bizarre.
9. Figure 1 main text and Figure 2 appendix L.2 are missing colour legends.
10. Appendix L.3 Fig 6, unclear to me what is meant by assumed sequencing error. Did you let both The Real McCOIL and coiaf infer the error? That would seem the fairest way to compare the two approaches.
